# Diversity and genetic architecture of agro-morphological traits in a core collection of European traditional tomato

**DOI:** 10.1093/jxb/erad306

**Published:** 2023-08-01

**Authors:** Clara Pons, Joan Casals, Matthijs Brower, Adriana Sacco, Alessandro Riccini, Patrick Hendrickx, Maria del Rosario Figás, Josef Fisher, Silvana Grandillo, Andrea Mazzucato, Salvador Soler, Dani Zamir, Mathilde Causse, Maria José Díez, Richard Finkers, Jaime Prohens, Antonio Jose Monforte, Antonio Granell

**Affiliations:** Instituto de Conservación y Mejora de la Agrodiversidad Valenciana (COMAV), Universitat Politècnica de València, València, Spain; Instituto de Biología Molecular y Celular de Plantas (IBMCP). Consejo Superior de Investigaciones Científicas (CSIC), Universitat Politècnica de València, València, Spain; Department of Agri-Food Engineering and Biotechnology/Miquel Agustí Foundation, Universitat Politècnica de Catalunya, Campus Baix Llobregat, Esteve Terrades 8, 08860 Castelldefels, Spain; Wageningen University & Research, Plant Breeding, POB 386, NL-6700 AJ Wageningen, The Netherlands; Institute of Biosciences and BioResources (IBBR), National Research Council of Italy (CNR), Via Università 133, 80055 Portici, Italy; Department of Agriculture and Forest Sciences (DAFNE), Università degli Studi della Tuscia, Viterbo, Italy; Wageningen University & Research, Plant Breeding, POB 386, NL-6700 AJ Wageningen, The Netherlands; Instituto de Conservación y Mejora de la Agrodiversidad Valenciana (COMAV), Universitat Politècnica de València, València, Spain; Hebrew University of Jerusalem, Robert H. Smith Institute of Plant Sciences and Genetics in Agriculture, Rehovot, Israel; Institute of Biosciences and BioResources (IBBR), National Research Council of Italy (CNR), Via Università 133, 80055 Portici, Italy; Department of Agriculture and Forest Sciences (DAFNE), Università degli Studi della Tuscia, Viterbo, Italy; Instituto de Conservación y Mejora de la Agrodiversidad Valenciana (COMAV), Universitat Politècnica de València, València, Spain; Hebrew University of Jerusalem, Robert H. Smith Institute of Plant Sciences and Genetics in Agriculture, Rehovot, Israel; INRAE, UR1052, Génétique et Amélioration des Fruits et Légumes 67 Allée des Chênes, Domaine Saint Maurice, CS60094, Montfavet, 84143, France; Instituto de Conservación y Mejora de la Agrodiversidad Valenciana (COMAV), Universitat Politècnica de València, València, Spain; Wageningen University & Research, Plant Breeding, POB 386, NL-6700 AJ Wageningen, The Netherlands; Instituto de Conservación y Mejora de la Agrodiversidad Valenciana (COMAV), Universitat Politècnica de València, València, Spain; Instituto de Biología Molecular y Celular de Plantas (IBMCP). Consejo Superior de Investigaciones Científicas (CSIC), Universitat Politècnica de València, València, Spain; Instituto de Biología Molecular y Celular de Plantas (IBMCP). Consejo Superior de Investigaciones Científicas (CSIC), Universitat Politècnica de València, València, Spain; University of Trento, Italy

**Keywords:** Agro-morphological traits, core collection, G×E, GWAS, haplotype, multi-environment trial, QTL, traditional tomato

## Abstract

European traditional tomato varieties have been selected by farmers given their consistent performance and adaptation to local growing conditions. Here we developed a multipurpose core collection, comprising 226 accessions representative of the genotypic, phenotypic, and geographical diversity present in European traditional tomatoes, to investigate the basis of their phenotypic variation, gene×environment interactions, and stability for 33 agro-morphological traits. Comparison of the traditional varieties with a modern reference panel revealed that some traditional varieties displayed excellent agronomic performance and high trait stability, as good as or better than that of their modern counterparts. We conducted genome-wide association and genome-wide environment interaction studies and detected 141 quantitative trait loci (QTLs). Out of those, 47 QTLs were associated with the phenotype mean (meanQTLs), 41 with stability (stbQTLs), and 53 QTL-by-environment interactions (QTIs). Most QTLs displayed additive gene actions, with the exception of stbQTLs, which were mostly recessive and overdominant QTLs. Both common and specific loci controlled the phenotype mean and stability variation in traditional tomato; however, a larger proportion of specific QTLs was observed, indicating that the stability gene regulatory model is the predominant one. Developmental genes tended to map close to meanQTLs, while genes involved in stress response, hormone metabolism, and signalling were found within regions affecting stability. A total of 137 marker–trait associations for phenotypic means and stability were novel, and therefore our study enhances the understanding of the genetic basis of valuable agronomic traits and opens up a new avenue for an exploitation of the allelic diversity available within European traditional tomato germplasm.

## Teaser

A multipurpose core collection of European traditional tomatoes has been established and analysed, revealing QTLs associated with agro-morphological trait stability and their mode of inheritance to exploit this genetic resource.

## Introduction

Investigating the genetic basis of complex traits is key for crop breeding. In the last 30 years, extensive quantitative trait locus (QTL) mapping and genome-wide association studies (GWAS) and their meta-analyses have resulted in a better understanding of the genetic basis of yield and associated traits, plant architecture, fruit appearance, and quality traits of tomato (Paran and van der Knaap, 2007; Ariizumi *et al.*, 2013; Monforte *et al.*, 2014; Rothan *et al.*, 2019; Martina *et al.*, 2021); consequently, a large number of genes involved in agronomic traits have been identified (Paran and van der Knaap, 2007; Ariizumi *et al.*, 2013; Monforte *et al.*, 2014; Rothan *et al.*, 2019; Martina *et al.*, 2021). However, our understanding of how genotypes respond to different environments is still limited. Two terms describe the degree of sensitivity of a genotype to the environmental variation: stability (Waddington, 1942) and phenotypic plasticity (Bradshaw, 1965). Stability is the ability of genotypes to buffer their developmental processes against environmental fluctuations (Waddington, 1942). Phenotypic plasticity, in contrast, is defined as the ability of a genotype to display different phenotypes as a response to different environments (Bradshaw, 1965). When variation in plasticity exists among different genotypes, it is termed genotype by environment interaction (GEI) (Monforte, 2020).

Understanding the extent of how the environment can modify the phenotype of a trait is of great interest in plant breeding. From an applied point of view, the appropriate degree of plasticity depends on the breeding objective (Monforte, 2020). A high stability to environmental fluctuations and consistent performance of genotypes are some of the main objectives in breeding programmes for wide adaptation, especially under the current situation of climate change (Kusmec *et al.*, 2018). However, a high trait plasticity is still needed in breeding programmes to obtain cultivars adapted to specific environments (Bernardo, 2020).

Most of the studies directed at investigating the phenotypic response of tomato (*Solanum lycopersicum* L.) to the environment have been conducted on experimental populations exposed to only two conditions (i.e. control versus stress) (Diouf *et al.*, 2020), such as water stress and/or salinity (Villalta *et al.*, 2007; Albert *et al.*, 2016; Diouf *et al.*, 2018) and heat response (Grilli *et al.*, 2007; Lin *et al.*, 2010; Xu *et al.*, 2017; Driedonks, 2018; Ruggieri *et al.*, 2019; Gonzalo *et al.*, 2020). However, while controlled stress trials are common in academia, plant breeders usually screen GEI using ‘multi-environment trials’ (METs), which provide a more realistic scenario of crop cultivation. In a MET, a number of genotypes are evaluated at several geographical locations and/or on different years, under the assumption that their phenotypic response would be representative of their response to future environments (Malosetti *et al.*, 2013).

Via *et al*. (1995) proposed two classes of genetic effects that influence plastic responses to the environment: (i) some alleles may be differentially expressed among environments, with varying effects on the phenotype (‘allelic sensitivity’); and (ii) regulatory loci may cause other genes to be turned on or off in particular environments (‘gene regulation’). These models are neither mutually exclusive nor make restrictions regarding the types of genes expected to be acting under each model (Ungerer *et al.*, 2003). Also, intra-locus (dominance) and inter-loci (epistasis) allelic interactions, as well as epigenetics, genetic linkage, and genome duplication/redundancy, can contribute to variation of stability among genotypes, as well as increase the potential for fine-tuning development and environmental responses, and generating new trait variation (El-Soda *et al.*, 2014; Lachowiec *et al.*, 2016). Recent studies have been undertaken to associate chromosome regions with trait stability/plasticity in tomato and other crops, by modelling stability as a trait, or modelling QTL by environment interactions (QTIs) using experimental mapping populations. These studies have demonstrated that plasticity and trait means are controlled by both specific and common genetic bases (Alseekh *et al.*, 2017; Kusmec *et al.*, 2017, 2018; Diouf *et al.*, 2020; Fisher and Zamir, 2021). In maize, allelic sensitivity seems to be the main driver of plasticity, and loci associated with GEI were predominant in regulatory regions of gene-proximal regions (Gage *et al.*, 2017). In tomato, the gene regulatory model was predominant for stability of both agronomic (Diouf *et al.*, 2020) and metabolite traits (Alseekh *et al.*, 2017), although co-localization of some QTLs and QTIs indicates that allelic sensitivity and genetic linkage mechanisms may contribute to plasticity in this crop (Diouf *et al.*, 2020). Moreover, in the case of metabolic stability, most of the QTLs were usually not associated with known metabolism genes, but rather with regulatory genes (Alseekh *et al.*, 2017), supporting the gene regulatory model. Most interestingly, a large number of stability QTLs for different metabolic or agronomic traits co-localize to the same region of chromosomes 10 and 11, respectively (Alseekh *et al.*, 2017; Diouf *et al.*, 2020), suggesting that environmental trait variation can be buffered by common regulatory genomic regions harbouring a pleiotropic gene or stability gene modules.

Whilst tomato plasticity/stability QTLs obtained using interspecific mapping populations may have been selected during tomato domestication and early diversification (Diouf *et al.*, 2020), the study of plasticity QTLs segregating in the European traditional tomato gene pool may identify those selected by farmers during the 500 years of cultivation in the Southern European Region, the secondary centre of tomato diversification (Villand *et al.*, 1998; Mazzucato *et al.*, 2008; Blanca *et al.*, 2022). In contrast to wild species or mapping populations, a traditional variety is the result of complex past and contemporary genetic exchanges, natural and farmer-mediated selection, adaptation to the pedoclimatic conditions, traditional management, and uses (Casañas *et al.*, 2017). A local adaptation could limit the response of traditional varieties to future environmental variations such as climate change, new environments, and/or new agricultural conditions and systems (Corrado and Rao, 2017). Alternatively, locally adapted varieties could be a source of genetic diversity for traits and their stability/plasticity, to be used in breeding programmes and modern sustainable agriculture (Casañas *et al.*, 2017).

During the last decades, a renewed interest has been observed in traditional varieties [vintage, landraces, and heirlooms are considered synonymous with traditional (Blanca *et al.*, 2022)], given their recognized value as a reservoir of genes, and the change in society’s interest for the environment, locally produced food, and identity issues (Casañas *et al.*, 2017). Despite the above, trait stability in traditional tomato has seldom been addressed. Most studies have focused on the adaptation of a few European traditional tomato varietal types to modern cultivation systems (i.e. low inputs versus high inputs, open field versus greenhouse, etc.) (Figàs *et al*., 2018a, b; Casals *et al.*, 2021) to increase their competitiveness against high-input cultivated modern varieties (Casañas *et al.*, 2017). The study of phenotypic responses to environment and GEI in European traditional tomato would provide insights into the stability and the adaptation of particular varietal types to modern cultivation systems. Nevertheless, to investigate the genetic mechanisms regulating GEI of agronomic traits, and to exploit the allelic diversity available within the European traditional germplasm, it is necessary to cover the full genetic diversity of the European traditional tomato pool. Traditional tomato presents a lower allelic diversity compared with contemporary varieties (Corrado *et al.*, 2014; Blanca *et al.*, 2022), although the former may be richer in rare alleles due to low breeding activity (Tripodi *et al.*, 2021). Despite this low genetic variability, panels of traditional varieties have proved sufficient for GWAS identifying numerous associations and novel variability (Ruggieri *et al.*, 2014; Sacco *et al.*, 2015; Baldina *et al.*, 2016; Blanca *et al.*, 2022; Pons *et al.*, 2022).

Recently, we have characterized a European traditional tomato collection consisting of 1489 genotypes (TRADITOM collection) to gain insights into the architecture of phenotypic variation (Pons *et al.*, 2022). However, its large size made it difficult to handle for METs and therefore for GEI analysis. Core collections (limited sets of entries derived from the whole genetic pool that represents the total collection’s diversity; Frankel, 1984) have been demonstrated to be useful for the efficient and economical utilization of plant germplasm and have been demonstrated to be useful as GWAS panels (Wang *et al.*, 2011; Sauvage *et al.*, 2014; Cao *et al.*, 2016; Sokolkova *et al.*, 2020), capturing the associations prevalent in the original collections (Jeong *et al.*, 2019; Kumar *et al.*, 2020). We have therefore developed a multipurpose core collection (TCC), comprising 226 European traditional tomato accessions from the original TRADITOM collection. The TCC captured most of the genotypic and phenotypic variation and geographical origin present in European traditional tomato. The TCC together, with a collection of 39 modern varieties equivalent to the traditional tomato types, was used to analyse 33 relevant agronomic traits and evaluate their stability across four independent locations. Our aim is to decipher the genetic control of the response to environmental variation in European traditional tomato and compare the response of traditional varieties with that of modern cultivars and inbreds.

## Materials and methods

### Development of a traditional core collection

To establish a core collection, accessions were selected using a mixed approach based on genotyping, phenotyping, geographic data, and expert knowledge. For genetic data, we have compiled and merged single nucleotide polymorphism (SNP) genotyping data that were previously published from 1850 tomato accessions. These comprised 1342 TRADITOM traditional accessions (Blanca *et al.*, 2022; Pons *et al.*, 2022), complemented with the same subset of SNPs selected from 150 re-sequenced accessions (Aflitos *et al.*, 2014), eight parents from a tomato MAGIC population (Causse *et al.*, 2013), and 350 accessions from an additional re-sequencing initiative (Lin *et al.*, 2014). These last three collections contained accessions collected principally in America (mainly South America), Europe (predominantly Italy), and Asia (mostly Russia) comprising *Solanum lycopersicum* accessions, as well as semi-wild (*S. lycopersicum* var. *cerasiforme*) and wild relatives such as *S. pimpinellifolium*, *S. arcanum*, *S. habrochaites*, *S. pennellii*, *S*. *galapagense*, *S*. *cheesmaniae*, *S*. *chmielewskii*, *S. neorickii*, *S. peruvianum*, *S. corneliomulleri*, *S. chilense*, and *S. habrochaites*. The initial SNP dataset containing 138 839 common markers was quality control checked and filtered. Plink, version 1.90 (https://www.cog-genomics.org/plink2) was used for initial filtering and pre-processing of the data. As 139 000 SNP markers is an overkill excessive for the expected variation, a random sample of 20% of the markers was taken. Genotypes with >30% missing data points were removed, as were markers with >10% of missing data points. The remaining missing genotype scores were imputed by assigning the score of the major occurring allele to that data point. The Adegenet R package (Jombart, 2008; Jombart and Ahmed, 2011) and principal coordinates analysis (PCoA) were used to quantify the amount of variability within and between the tomato accessions. Traditional accessions were selected to optimize the genetic diversity, based on the first three axis of the PCoA biplot for optimizing the selection of accessions within the core collections, by both genotypic and phenotypic diversity (Odong *et al.*, 2013; van Heerwaarden *et al.*, 2013)

For phenotypic diversity, we used data from 67 traits previously phenotyped in 1489 TRADITOM accessions (Pons *et al.*, 2022). Phenotypic selection was done by selecting 20 varieties showing extreme phenotypes for quantitative traits (10 highest and 10 lowest), and a subset of random varieties selected for each category within a qualitative trait. To further ensure that the original geographic distribution was represented in the core collection, traditional tomato entries selected from phenotypic and genotypic data were plotted per province based on passport data (Pons *et al.*, 2022) in a Google map to identify geographical regions where no accession was selected. Accessions without seed stock or those that showed phenotypic segregation in field trials were discarded from the collection and replaced by accessions that tightly clustered in the PCoA biplot, considering expert knowledge aspects (popularity, prestige, role in breeding history, or presence of phenotypic features of interest).

### Core collection evaluation

Tassel v5.0 (Bradbury *et al.*, 2007) was used to calculate genetic diversity indexes, minor allele frequency (MAF), kinship matrix, and multi-dimensional scaling (MDS). Nucleotide diversity (π) was calculated for each SNP. Pairwise kinship coefficients were calculated using centred identity-by-state (IBS)-based method (Endelman and Jannink, 2012). MDS analysis was based on pairwise IBS distances. ggplot2 (Wickham, 2016) and plotly (Sievert, 2020) R packages were used to generate plots. The number of SNPs located within bins of 1 Mb were plotted by SRPlot (https://www.bioinformatics.com.cn/en), a free online platform for data analysis and visualization. Violin plots and Wilcox test were used to assess differences in quantitative trait distribution. Mosaic plots, χ^2^ independence test, and standardized Pearson’s residuals (*d*_ij_) were used to evaluate qualitative variable distribution and class coverage. ggplot2 (Wickham, 2016) and vcd (Meyer *et al.*, 2006) R packages were used to generate plots. All the statistical tests were considered significant with a *P*-value <0.001.

### Modern collection

A modern tomato collection consisting of 39 modern cultivars was created by selecting hybrids and inbred breeding lines from commercial companies and breeding institutes. Selected modern tomatoes represented tomatoes cultivated in Southern Europe and belonging to the same typologies of traditional tomatoes in terms of biochemical composition, sensory profiles, and consumer preferences (Sinesio *et al.*, 2021). These modern cultivars in some cases are replacing the traditional cultivars in their regions of cultivation.

### Plant phenotyping collection

The traditional core collection and the modern tomato collection were phenotyped in the experimental fields of COMAV-UPV (Spain) and UNITUS (Italy) during spring–summer 2016, and in INRA (France) and HUJI-ARO (Israel) experimental fields during spring–summer 2017, by using their current cultivation practices (for details of cultivation practices, see Supplementary Table S1). Three plants per accession were grown in a randomized design at each location. The phenotyped traits included quantitative and qualitative traits related to agronomical performance, flowering and ripening precocity, fruit quality, fruit shape, incidence of physiological disorders, and plant and inflorescence architecture (Supplementary Table S2). Detailed information about the phenotyping procedure and scoring is provided in Supplementary Table S2. All traits were scored as a unique observation per accession, excepting mean fruit weight (g) on trusses 1–4 (FW1.4), mean commercial fruit weight (g) on trusses 1–4 (FW1.4c), mean non-commercial fruit weight (g) on trusses 1–4 (FW1.4nc), total estimated yield per plant (EstYIELD), commercial yield on trusses 1–4 (Yield1.4c), non-commercial yield on trusses 1–4 (Yield1.4nc), total yield on trusses 1–4 (YIELD1.4T), number of commercial fruits on trusses 1–4 (FN1.4c), number of non-commercial fruits on trusses 1–4 (FN1.4nc), total number of fruits on trusses 1–4 (TNF1.4), total number of fruits per plant (TNFP), and the CIELAB colour coordinates L*, a*, b*, the chroma (C*), and the hue angle (H_ab_) which were recorded in each plant.

Pre-processing of trait data was performed according to Pons *et al.* (2022). The heatmap of raw data was generated using clustvis (Metsalu and Vilo, 2015). Rows were centred; unit variance scaling was applied to rows. Rows were clustered using correlation distance and Ward linkage.

### Analysis of variance

The phenotypic variance of traits that were recorded on a plant basis was partitioned into components due to the variation of genotype (G), location, or environment (E), and their interaction (GEI). The ANOVA was performed in metan (Olivoto and Dal’Col Lúcio, 2020), fitting each trait to a mixed linear model in a combined ANOVA across environments as follows:


$${\text{Y}}_{\text{ijk}}=\;\mu \;+{\text{G}}_{i}+{\text{E}}_{j}+\;\;\;{\left(\text{G}\times \text{E}\right)}_{\text{ij}}+\;\varepsilon \text{​}{\;}_{\text{ijk}}$$ (1)

Where Y_ijk_ is the response variable of the kth replicate of the ith genotype in the jth location (i=1–260 accessions; j=1–4 locations; k=1–3 plants); μ is the grand mean; G_i_ is the main effect of the ith genotype; E_j_ is the main effect of the jth location; (G×E)_ij_ is the interaction effect of the ith genotype with the jth location; and ε_ijk_ is the random error distributed. ANOVA and variance components estimations were performed using a linear mixed model using residual maximum likelihood, taking the expected genotype means μ as a fixed effect and the rest of factors as random, assuming G_i_~NID (0, σ_g_^2^); E_j_ ~NID (0, σ_E_^2^); (G×E)_ij_~NID (0, σ_ge_^2^); ε_ijk_~NID (0,σ^2^), where NID means normally, identically, and independently distributed. The statistical significance of variance components was estimated by a likelihood ratio test, and probability was obtained by a two-tailed χ^2^ test with one degree of freedom. The best linear unbiased predictors (BLUPs) per variety across environments (BLUPg) and per variety for the interaction G×E (BLUPge), and broad sense heritability (H^2^) were obtained employing a linear mixed model using both mean and environment effect as fixed, and genotype and interaction as random variables. Broad sense heritability was estimated across environments as:


$${\text{H}}_{g}^{2}=\frac{\hat{\sigma }{\;}_{g}^{2}}{{\hat{\sigma }}_{\text{g}}^{2}\text{+}{\hat{\sigma }}_{\text{ge}}^{2}\text{+}\hat{\sigma }\text{​}{\;}_{\epsilon }^{2}}$$ (2)

In the case of the traits collected on a per accession mean basis (qualitative traits and quantitative traits related to flowering and ripening precocity and physiological disorders), the effects of environment and genotype were estimated in R (R Development Core Team, 2018) using type III of a two-way ANOVA with fixed effects and without interaction. GEI was assessed by interaction plots using the interaction.plot function of R.

### Stability indexes

Stability indexes were calculated in metan (Olivoto and Dal’Col Lúcio, 2020) for accessions where the trait was recorded in at least three trials. For quantitative traits with three plants measured per accession, stability was estimated by the weighted average of absolute scores (WAASB) (Olivoto *et al.*, 2019) from the singular value decomposition of the matrix (or interaction principal components axis, IPCA) of BLUPs for the GEI effects generated by the random model described above as follows:


$${\text{WAASB}}_{\text{i}}=\sum_{\text{k}=1}^{\text{p}}\left|{\text{IPCA}}_{\text{ik}}\times {\text{EP}}_{\text{k}}\right|/\sum_{\text{k}=1}^{\text{p}}{\text{EP}}_{\text{k}}$$ (3)

where WAASB_i_ is the weighted average of absolute scores of the ith genotype, IPCA_ik_ is the score of the ith genotype in the kth IPCA, and EP_k_ is the amount of the variance explained by the kth IPCA. The genotype with the lowest WAASB value was considered the most stable, that is, the one that deviates least from the average performance across locations.

In the case of traits collected on a per accession mean basis, the stability index was estimated as the projection of the genotype i onto the average environment coordination (AEC) ordinate (Yan, 2001; Yan and Tinker, 2006), on the genotype main effects GEI model (GGE model) biplot (Yan, 2001; Yan and Kang, 2002). Briefly, the GGE model for a trait mean of genotype i in environment j (Y_ij_) can be written as:


$${\text{Y}}_{\text{ij}}=\;\mu \;+{\text{E}}_{j}+\sum_{\text{k}=1}^{\text{K}}\;\lambda \text{​}{\;}_{k}\;\xi \text{​}{\;}_{\text{ik}}\;\eta \text{​}{\;}_{\text{jk}}+\epsilon_{\text{ij}}$$ (4)

Where µ is the grand mean; E_j_ is the location j environmental main effect; ε_ij_=residual effect~N (0,σ^2^); λ_k_=the singular decomposition value (eigenvalue) for the component (PC); ξik=the eigen-vector of genotype i for PC_*k*_; η_jk_=the eigen-vector of environment j for PC_k_; K is the number of PC axes retained in the model [K≤min (g,e) and K=2 for a two-dimensional biplot]; and ε_ij_=the residual associated with genotype i in location j. For stability estimation, the mean of PC1 and PC2 scores for all locations calculated using genotype‐focused singular value partitioning (SVP) was used to define an average environment. The line passing through this average location and the biplot origin serves as the abscissa of the AEC and represents the mean genotype. The ordinate of the AEC is the line that passes through the origin and is perpendicular to the AEC abscissa and approximate to the GEI associated with each genotype. The length of the projection of a genotype onto the AEC ordinate (P^AEC^_i_), regardless of the direction, is an estimation of stability. The more stable the genotype, the shorter the projection onto the AEC ordinate.

Violin plots depicting variability in trait stability were constructed in gg2plot (Wickham, 2016) using unit variance scaled data. Pearson correlations between mean (in the case of traits scored as unique observation per accession) and BLUPg (in the case of quantitative data with three plants measured per accession), and stability indexes were performed using the corrplot package (Wei, 2017). The agglomeration complete method was used for correlation clustering. Only correlations with significant levels <0.01 were plotted.

### Stability GWAS and genome-wide by environment interaction studies

The 111 110 SNPs generated by genome by sequencing (GBS) in Blanca *et al.* (2022) and Pons *et al.* (2022) for the TCC accessions studied here were used for association analysis. Filtering was performed by ensuring a maximum of 25% missing markers for each individual, a maximum of 25% missing individuals for each marker, an MAF of 5%, and a homozygous fraction <5%. To identify SNPs associated with the mean, GEI, and stability, two approaches were used.

To model stability–SNP associations, a GWAS was performed using a linear mixed linear model including the co-ancestry kinship matrix among genotypes as a random effect:


Y=X β +g+e (5)

Where Y is an *n*×1 vector of the estimated stability index vector, X is the molecular marker matrix with *n*×p dimensions, β is the unknown *n*×1 vector of allelic effects to be estimated, g is an *n*×*n* co-ancestry kinship matrix, and e is the random error *n*×1 vector. The GWASs were performed using the R package EMMA (Kang *et al.*, 2008). The kinship matrix was an identity by state matrix calculated by using the function emma.kinship, with an additive model for heterozygous alleles.

To model trait means and variance (GEI term), we performed a two-stage approximation. First, we estimated the mean across locations and the GEI effects using a linear fixed effect model:


$${\text{Y}}_{\text{ij}}=\;\mu \;\ldots +{\text{G}}_{i}+{\text{E}}_{j}+\;\;\;{\left(\text{GE}\right)}_{\text{ij}}+\;\varepsilon \text{​}{\;}_{\text{ij}}$$ (6)

Where Y_ij_ is the adjusted phenotypic value of the ith genotype in the jth location (i=1, 2, … , g; j=1, 2, … , e); μ is the estimated grand mean; G_i_ is the main effect of the ith genotype; E_j_ is the main effect of the jth location; (GE)_ij_ is the interaction effect of the ith genotype with the jth location; and ε_ij_ is the random error distributed. Then, we performed the GWAS on the estimated trait means and GEI effects vectors following a linear mixed model including the co-ancestry kinship matrix among genotypes as a random effect as described above.

For both models, the significant threshold for GWAS results was adjusted for multiple testing using the effective number of independent tests (Li and Ji, 2005).

### Single nucleotide polymorphism candidate region, loci, and quantitative trait locus definition

The confidence region for each significant SNP from GWAS and genome-wide by environment interaction studies (GWEIS) was defined on a window size defined by the SNPs with an intervariant allele count squared correlations (*r*^2^) higher than the interchromosomal linkage disequilibrium (LD) at the 95th percentile (*r*^2^ baseline value >0.4456) and within a distance <2 Mb. Loci were defined by merging lead SNP candidate regions that physically overlapped and were found in an associated LD block as follows: for each lead SNP, regional LD was calculated within the candidate gene region using the imputed SNP matrix (Pons *et al.*, 2022). LD blocks were defined for SNPs with *r*^2^ higher than the baseline *r*^2^ value. Overlapping LD blocks or close (within 100 kb) were combined to conform associated loci using beddtools (Quinlan and Hall, 2010). SNPs significantly associated with a specific trait and located within the same locus collectively constitute a single QTL.

The selection of candidate genes within the confidence region was based on gene description, gene ontology, and relevant research papers on gene functions and expression databases.

### Estimating meanQTL and stbQTL mode of inheritance

The mode of inheritance of meanQTL and stbQTL GWAS significant SNPs was tested in a two-step procedure. First the effect of the allelic state of a given SNP (AA, AB, BB for each individual: A for reference allele, and B for alternative allele) on the expression of a trait was modelled using the following linear models using the lm4 R package (Bates *et al.*, 2015):

In the case of the trait mean associations:


$${\text{BLUPg}}_{\text{i}}=\;\beta \text{​}{\;}_{0}+\;\beta \text{​}{\;}_{1}{\text{SNP}}_{\text{i}}+\;\varepsilon \text{​}{\;}_{\text{ij}}$$ (7)

Where BLUPg_i_ is the BLUP of thephenotypic value of the ith variety (i=1, 2, … , g) across environments obtained from the ANOVA model, with intercept β_0_; β_1_ the marker effect; SNP_i_ a vector of the marker allelic states; and ε_ij_ the random error distributed.

In the case of the stability index associations:


$${\text{Stab}}_{\text{i}}=\;\beta \text{​}{\;}_{0}+\;\beta \text{​}{\;}_{1}{\text{SNP}}_{\text{i}}+\;\varepsilon \text{​}{\;}_{\text{ij}}$$ (8)

Where Stab_i_ is the stability index (WAASB or P^AEC^_i_) of the trait in the ith variety (i=1, 2, … , g) across environments; with intercept β_0_; β_1_ the marker effect; SNP_i_ a vector of the marker allelic states; and ε_ij_ the random error distributed.

Then, marginal means for the different marker allelic states were extracted from the linear models using the R package emmeans (Lenth *et al.*, 2019) taking into account only the SNP effect for those allelic states shared by >3 accessions. Pairwise multiple comparisons between the marginal means for the different marker allelic states were used to test differences in the expression of a trait or stability index between allelic states. Marginal means for the different marker allelic states were then tested for an additive mode of action using the following contrast where AA and BB are the marginal phenotypic mean values of the homozygous genotypes, and AB is the marginal phenotypic mean value of the heterozygous genotype. If the null hypothesis of the additive contrast is rejected, then the following contrasts are performed to test dominant or recessive modes of action.

If B has a positive effect (BB>AA)


$$\begin{array}{c} \text{Ho}:\text{AB}-\text{BB}=0;\text{dominant }\;\text{model}\\ \text{Ho}:\text{AB}-\text{AA}=0;\text{recessive }\;\text{ model} \end{array}$$

If B has a negative effect (AA>BB)


$$\begin{array}{c} \text{Ho}:\text{AB}-\text{AA}=0;\text{dominant }\;\text{ model}\\ \text{Ho}:\text{AB}-\text{BB}=0;\text{recessive }\;\text{ model} \end{array}$$

When all models are rejected and the AB is higher than both AA and BB, then it is considered overdominance.

Where the additive genotypic value is:


$$\text{a}=\frac{\text{AA}-\text{BB}}{2}$$ (9)

and the dominance genotypic value is:


$$\text{d}=\text{AB}-\frac{\text{AA}+\text{BB}}{2}$$ (10)

### Mode of action and QTI interaction type

To study the mode of inheritance of significant QTI of quantitative traits, the allelic state effect of a given SNP on the expression of a trait at individual environmental combinations was modelled using the following linear models utilizing the lm4 R package (Bates *et al.*, 2015):


$${\text{BLUPge}}_{\text{ij}}=\;\beta \text{​}{\;}_{0}+\;\beta \text{​}{\;}_{1}{\text{SNP}}_{\text{i}}+{\text{E}}_{j}+\;\;\;{\left(\text{SNP}\times \text{E}\right)}_{\text{ij}}+\;\varepsilon \text{​}{\;}_{\text{ij}}$$ (11)

Where BLUPge_ij_ is the best linear unbiased estimation for genotype–location interaction of phenotypic value in the ith accession (i=1, 2, … , g) in the jth location obtained from the model in Equation 11; β_0_ is the global intercept; β_1_ is the marker effect; SNP_i_ is a vector of the marker allelic state; (SNP×E)_ij_ is the interaction effect of the ith marker genotype with the jth location; and ε_ij_ is the random error distributed.

Then, marginal means for the different marker allelic states were extracted from the linear models using the R package emmeans (Lenth *et al.*, 2019) taking into account the SNP×E interaction effect for those allelic states shared by >3 accessions. Pairwise multivariate comparisons were performed via the Mahalanobis distance between one set of four means and another, and Hotelling’s *T*^2^ statistics using the mvcontrast function. Additivity, recessivity, and dominance were tested using the contrast described above.

To study the direction of effect and the QTI interaction type, differences between the marginal means of BLUP_ge_ for the two homozygous marker allelic states at individual location combinations were compared using a one-way ANOVA. Location-specific allelic effects were classified in three QTL types according to El-Soda *et al.* (2014): (i) QTIs where the effect on the phenotype is in the same direction but differs in the magnitude from one environment to another (QTL3); (ii) QTIs that are conditionally neutral, where the effects are only detected in some environments but not in others (QTL4); (iii) and QTIs with opposite phenotypic effects when comparing different environments (QTL5).

The mode of action and allelic effect direction were considered consistent for a given QTL when those estimates were similar for all the SNPs included in that QTL. In the case of different values, these estimates were considered as non-conclusive.

### Linkage disequilibrium and haplotype analyses

Haploview version 4.1 (Barrett *et al.*, 2005) was used to generate the LD plot and to define haplotype blocks based on Lewontin’s normalized LD (*Dʹ*) value for all pairwise combinations of SNPs spaced <1 Mb within the region of interest. The LD and haplotype analysis were performed using Haploview default parameters (MAF <0.001, Hardy–Weinberg equilibrium test <0.001, and missing values <75%). Haplotype blocks partitioning (i.e. segments of consistently high *Dʹ* that break down where high recombination rates, recombination hotspots, and obligate recombination events (Myers and Griffiths, 2003) was performed using the solid spine method.

The haplotype–trait association was analysed by a two-way ANOVA model in Rstudio (Racine, 2012) as follows:


$${\text{Y}}_{\text{ijk}}=\;\mu \;+{\text{H}}_{i}+{\text{C}}_{j}+\;\;\;{\left(\text{H}\times \text{C}\right)}_{\text{ij}}+\;\varepsilon \text{​}{\;}_{\text{ijk}}$$ (12)

Where Y_ijk_ is the trait value of the kth accession of the ith haplotype in the jth collection (i.e. TCC or TRADITOM); μ is the grand mean; H_i_ is the main effect of the ith haplotype; C_j_ is the main effect of the jth collection; (H×C)_ij_ is the interaction effect of the ith haplotype with the jth collection; and ε_ijk_ is the random error distributed.

For fruit weight, pairwise haplotype genotype mean comparisons within collection and between collections were performed with Tukey’s honestly significant difference (HSD) test (*P*<0.05). The mean and the SD for each haplotype genotype in each collection were extracted from the ANOVA model using the R package emmeans (Lenth *et al.*, 2019). Box-plots were generated with ggplot2 R package (Wickham, 2016).

In the case of puffiness, a qualitative trait, haplotype–trait associations were evaluated with a χ^2^ test with Bonferroni false discovery rate (FDR) for pairwise nominal and ordinal comparisons of the proportions in each collection. Enrichment of phenotypic classes within genotypic classes was analysed using standardized Pearson’s residuals (*d*_ij_). The results were presented as a mosaic plot with the ‘vcd’ R package (Meyer *et al.*, 2006).

## Results

### Development of a multipurpose core collection in European traditional tomato

To create a core collection of European traditional tomato representative of 15–20% of the initial accessions (Brown and Spillane, 1999) in the TRADITOM collection, a mixed approach was used (see Supplementary Protocol S1 and Supplementary Figs S1 and S2A for more details). First, two sub-core sets of entries (accessions in the core collection) were selected. The first sub-core set consisted of 57 accessions aimed at optimizing genetic diversity. The second sub-core set comprised 133 entries, representing diversity in quantitative and qualitative phenotypic traits. Subsequently, 39 entries were selected to fill geographical gaps (Supplementary Fig. S2A). Thirty-five entries which either were not previously evaluated or exhibited segregation in preliminary field trials were replaced with accessions of historical relevance and tightly clustered to those selected in the first two sub-core sets (Supplementary Fig. S1). The final European traditional tomato core collection (TCC; Supplementary Table S3) comprised 226 entries, which accounted for 16.7% of the original collection. Within the TCC, 190 entries represent the genotypic, phenotypic, and geographical diversity cultivated in the Mediterranean basin between 1950 and 2015 (Blanca *et al.*, 2022; Pons *et al.*, 2022). Additionally, 35 accessions with historical relevance were included. Based on the classification by Blanca *et al.* (2022), 121 accessions in the TCC were classified as true vintage/landraces, 67 as ‘traditionalized’ (varieties classified as traditional based on their passport, but developed by local farmers from obsolete commercial varieties that contained resistance gene introgressions), and the remaining 38 were unclassified (Supplementary Table S3)

### Evaluation of the core collection

**Fig. 1.**
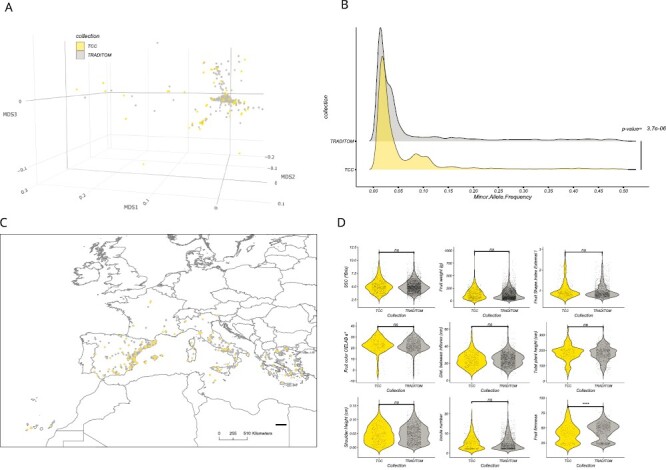
Comparison of genetic, phenotypic, and geographic diversity of the European traditional tomato core collection (TCC) with the initial TRADITOM collection. (A) Multidimensional scaling based on GBS data showing the accession in the entire TRADITOM collection and highlighting the 226 accessions comprising the TCC. (B) Minor allele frequency (MAF) distribution of the 5715 SNPs with MAF ≥0.01 compared with the distribution of the 9073 SNPs polymorphic in the TCC at MAF ≥0.01. The distributions are presented as densities. The *P*-value of the ANOVA test comparing distributions is shown. (C) Geographical distribution of TCC and of the remainder of the TRADITOM accessions. (D) Violin plots depicting the distribution for nine traits in the TCC and in the entire TRADITOM collection. Wilcox test results between TRADITOM and TCC are shown: ^ns^non-significant; *P≤0.05; **P≤0.01, *** *P*≤0.001, **** *P*≤0. 0001. The key indicates the collection: yellow TCC and grey TRADITOM.

The representativeness of the 226 entries of the TCC relative to the original TRADITOM collection was assessed comparing the diversity captured in both collections. This evaluation was based on passport, genotyping, and phenotyping datasets previously generated by Pons *et al.* (2022). MDS analysis, using genotypic data of 110 909 markers, showed that both collections exhibited a similar distribution of accessions in the MDS space, and the TCC covered the genetic variation of the entire TRADITOM (Fig. 1A). The percentage of SNP polymorphism, SNP distribution, MAF, and nucleotide diversity (π) parameters were, in general, equivalent between the entire TRADITOM and the TCC collections (see Supplementary Protocol S2, Fig. 1B, and Supplementary Figs S3 and 4). However, the TCC showed a 36.3% increase in π (π=0.0095, *P*-value <2.2e-16) with respect to TRADITOM, particularly on chr04, chr05, chr11, and chr12 (Supplementary Fig. S4B). The frequency of some rare alleles in the entire TRADITOM increased in the TCC (*P*-value=3.7e-6; Fig. 1B), which was also reflected in SNP density along chromosomes (Supplementary Fig. S3A).

The geographical distribution of entries was also similar between collections (Fig.1C; Supplementary Fig. S2B). Regarding phenotypic variation, violin plots showed that, in general, the means and the range of variation for the nine selected quantitative traits were similar among collections (*P*-value <0.01), with the exception of FIRM (Fig. 1D). For qualitative traits (Supplementary Fig. S5), TCC covered all the ranges of trait categories represented in the TRADITOM collection, although frequency distribution indicated non-homogeneity of distribution among the entire TRADITOM and TCC (Supplementary Fig. S5).

Population structure analysis using MDS (Supplementary Fig. S6A) and kinship coefficients matrix (Supplementary Figs S4C, S6C) indicated a low to moderate level of genetic relatedness among the majority of the TCC entries. Consequently, the TCC preserved the majority of variation of the entire TRADITOM and met the requirements for a core collection to be used as a GWAS panel (Kumar *et al.*, 2020).

### Phenotypic variance of agro-morphological traits in a multi-location trial

We conducted field experiments at four locations (Supplementary Table S1) to phenotype the TCC for 33 traits (Supplementary Table S2). In parallel, we phenotyped a reference set of 39 modern varieties, currently cultivated in Southern Europe and covering the same fruit typologies as the TCC varieties (Supplementary Table S4). The complete phenotyping dataset can be found in Supplementary Table S5.

Traditional, traditionalized, and modern groups showed significant (*P*<0.05) differences for most trait means. In brief, traditional tomatoes showed higher fruit weight (FW1.4c, FW1.4nc, and FW1.4) (Supplementary Fig. S7) and yield (YIELD1.4c, YIELD1.4n.c, EstYIELD, and YIELD1.4T) (Supplementary Fig. S8), but lower fruit size homogeneity (FSH), fruit set sequence (FSS), and number of fruits per plant (FN1.4c, TNF1.4, and TNFP) than traditionalized tomatoes (Supplementary Figs S7, S9). Further, traditional tomatoes in general had significantly longer time to flowering (FLOW) and ripening times (RIP), and lower ripening uniformity (RUN) than traditionalized tomatoes (Supplementary Fig. S10). In addition, traditional tomatoes had, on average, fruit with more irregular shapes (ITS), that were also more fasciated (FAS), softer (FIRM), and paler (fec.L*), but with a more intense red colour (fec.a*, fec.C*) and green shoulders (GSH) than traditionalized tomatoes (Supplementary Figs. S9, S11, S12). Incidences of puffiness (PUF), blossom end rot (BER), and radial cracking (RCRACK) were higher in traditional than in traditionalized tomatoes (Supplementary Fig. S12). Compared with the modern reference panel, the traditional tomatoes in general had a lower yield (YIELD1.4c, YIELD 1.4T, and EstYIELD), FSS, FSH, and FIRM, but a higher index for easiness to detach from the pedicel (EAS), longer FLOW and RIP times, and higher BER and PUF incidences (Supplementary Figs S7–S12). It is remarkable that some traditional varieties had similar or even higher yield than modern varietes (Supplementary Fig. S8). No significant differences in fruit weight and fruit number traits were found between traditional and modern tomatoes (Supplementary Fig. S7). The comparison between modern and traditionalized tomatoes showed that traditionalized ones had a significantly lower yield for all yield components, FN1.4nc, FW, FAS, and RCRACK but higher RIP, RUN, and BER (Supplementary Figs S7–S12).

**Fig. 2.**
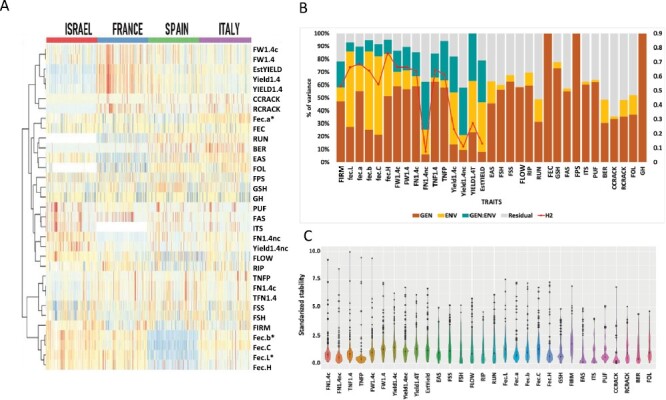
Phenotypic variability, sources of variation, and stability of 33 traits across four locations. (A) Hierarchical clustering of TCC based on the average trait value across the four locations. Traits are in rows and accessions in columns. The colour of the columns indicates the location. Trait means are centred, and scaled and clustered using correlation distance and Ward linkage. (B) Contribution of genetic, environmental, and interaction variances to the phenotypic variation and heritability across the four environments. Bar plots depict the proportion of variance due to genotype, environment, G×E, and residuals. The red line represents heritability. Traits are on the *x*-axis, percentage of variation on the primary *y*-axis, and heritability on the secondary *y*-axis. (C) Violin plots showing trait stability variation represented as unit variance standardized stability indexes. Inside the violin plots, the boxplots showing the median, first and third quartiles, and outliers (empty circle). FN1.4c, number of commercial fruits on trusses 1–4; FN1.4nc, number of non-commercial fruits on trusses 1–4; FW1.4, mean fruit weight (g) on trusses 1–4; FW1.4c, mean commercial fruit weight (g) on trusses 1–4; TNF1.4, total number of fruits on trusses 1–4; TNFP, total number of fruits per plant; Yield1.4c, commercial yield on trusses 1–4 (kg per plant); Yield1.4nc, non-commercial fruit yield on trusses 1–4 (kg per plant); YIELD1.4T, total yield on trusses 1–4 (kg per plant); EstYIELD, total estimated yield per plant (kg per plant); EAS, easiness of fruit to detach from the pedicel; FEC, fruit external colour; FPS, fruit predominant shape; GH, growth habit; FSH, fruit size homogeneity; FSS, fruit set sequence; FLOW, flowering earliness, number of days from sowing until 50% of plants have at least one open flower; RIP, ripening earliness, number of days from sowing until 50% of plants have at least one ripe fruit; RUN, ripening uniformity of the whole plot; fec.a*, CIELAB fruit external colour coordinate a; fec.b*, CIELAB fruit external colour coordinate b; fec.C*, CIELAB fruit external colour chroma; fec.H, CIELAB fruit external colour hue angle; fec.L*, CIELAB fruit external colour coordinate L; FIRM, fruit firmness; GSH, green shoulder; ITS, irregular transversal section; FAS, fruit fasciation; PUF, puffiness appearance; BER, blossom-end rot; CCRACK, presence and incidence of concentric cracking; RCRACK, presence and incidence of radial cracking; FOL, foliage density.

Hierarchical clustering of the phenotypic values for each accession in each location (Fig. 2A) grouped the traits into five clusters. This partition mainly reflected the patterns of trait variation across environments. The accession phenotypic values in each location (Fig. 2A) revealed differences in trait values across environments, with some of them, such as fruit colour parameters (fec.b*, fec.C*, fec.L*, and fec.H) and yield (EstYIELD, YIELD1.4c, and YIELD1.4T), easily identifiable. The phenotypic variances were decomposed into genotype (V_G_), environment (V_E_), and their interaction (V_GE_) variances (see the Materials and methods). We found highly significant V_G_ and V_E_ (*P*-value <0.001) for almost all the traits (Fig. 2B; Supplementary Table S6). Genetic variance ranged from 5.92% (FN1.4nc) to 99.87% (GH) of total variance. The environmental variance ranged from 0% (FEC, FPS, and GH) to 61.08% (fec.b*). Generally, V_G_ was larger than V_E_, except for some traits related to fruit colour (fec.L*, fec.b*, and fec.C*) and yield components Yield1.4c, YIELD1.4T, and EstYIELD. Similarly, V_GE_ was highly significant (*P*-value <0.001) for all 16 quantitative traits related to agronomical performance and fruit quality (Supplementary Table S6). The extent of GEI effect was, in general, lower than that of G and E in the case of fruit quality traits, while it was larger than that of E in the case of agronomical performance traits, being responsible for V_GE_ contributing from 7.01% (fec.L*) to 37.31% (FN1.4nc) of the total variance. The broad sense heritability across environments (H^2^) ranged from ~7% (FN1.4nc) to ~76% (fec.H) (Fig. 2B; Supplementary Table S6). In the case of traits for which only the accession mean was recorded (qualitative traits, physiological disorders, and flowering/ripening precocity), GEI could not be estimated (Malosetti *et al.*, 2013). However, the large values observed for residuals (Fig. 2B; Supplementary Table S6) and mean interaction plots (Supplementary Fig. S13) suggested the existence of GEI for these traits.

### Phenotypic stability and trait correlations in the different tomato groups

The significant GEI indicated that at least some of the accessions may have a low stability. The particular contribution of each accession to the GEI term was evaluated using stability indexes (i.e. the lower the values, the higher the stability; Fig. 2C; Supplementary Table S7; Supplementary Fig. S14). Violin plots (Fig. 2C) revealed variability in stability for all traits, with accessions characterized by having intermediate and low levels of stability. However, most of the genotypes (>50%) showed high stability estimates (<1) in 21 traits.

The stability and genotype average trait values showed, in general, moderate to strong correlations (significance *P*<0.001) for almost all the traits (Supplementary Fig. S15). In most cases, the least stable accessions produced the highest phenotypic values (i.e. the higher the phenotypic value, the higher the trait stability index). Only a few traits (fec.a*, FSH, and FSS) were negatively correlated with their stability index. Additionally, some traits were correlated with the stability of other traits. For example, FN1.4c, TNF1.4, and TNFP correlated with the stability for other agronomic traits such as traits related to physiological disorders and earliness classes (Supplementary Fig. S15A). Furthermore, the correlation analysis also revealed that (i) the more days to ripen (RIP), the less stable was the colour development of the fruit, and (ii) the higher the firmness (FIRM), the more stable the incidence of physiological disorders and the fruit shape. These correlations could be explained, at least in part, by the correlations existing between these traits (Supplementary Fig. S15B).

The range of variation for stability between traditional, traditionalized, and modern groups greatly overlapped (Supplementary Figs S7–S12), without statistically significant differences (*P*-value <0.05) for the stability of most of the traits. The only exceptions were fec.L*, FSH, FAS, ITS, CCRACK, BER, FLOW, and RUN (Supplementary Figs S9–S12), which showed significant differences in phenotypic stability between the modern and traditional tomatoes, while FAS and ITS did so between traditional and traditionalized groups (Supplementary Figs S10, S11), and fec.L* and FLOW between traditionalized and modern (Supplementary Figs S9, S12) groups. In these cases, the modern tomatoes showed, on average, the highest stability, while the traditional ones showed the lowest for this last group of traits. FSH, FLOW, RUN, and BER means (Supplementary Figs S9–S12) were also significantly different between traditional and modern tomatoes, while ITS and FAS were significantly different between traditional and traditionalized tomatoes (see above). The rest of the traits showing stability differences did not show differential phenotypic means as a function of the tomato group. Nevertheless, traditional varieties showed a wider range of trait stability than modern and traditionalized varieties (Supplementary Figs S8–S12).

### GWAS and GWEIS for phenotypes and stability

**Fig. 3.**
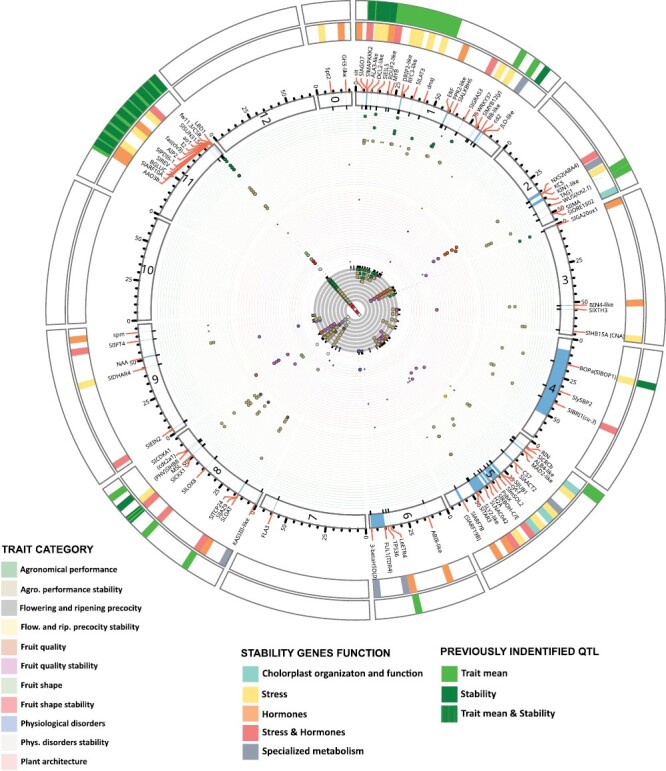
Overview of meanQTLs, stbQTLs, and QTIs across chromosomes. Fuji plot representing the 199 SNP–trait associations identified by GWAS and GWEIS in 86 SNPs located in 62 loci, their co-localization, candidate genes, functional category, and location of previously identified QTLs. The innermost ring (ring 1) represents the number of traits associated with each SNP. Rings 2–11 represent the genomic position of the SNP associated with trait, trait category, and the type of co-localization: larger dots indicate inter-categorical co-localization; medium dots, intra-categorical; and small dots, trait-specific association. Ring 12 represents chromosomes and linkage blocks around trait-associated SNPs. The two outer rings represent the main functional categories of candidate genes for stability and the location of previously reported QTLs for the same trait or its stability. The order of the traits in each trait category is (from the outer-most ring): agronomical performance: EAS, FN1_4c, FN1_4nc, FSH, FW1_4, FW1_4c, TNF1_4, TNFP, Yield1_4nc; agronomical performance stability: EstYIELD_QTI, FN1.4c_stab, FN1.4nc_QTI, FN1.4nc_stab, FSS.QTI, FW1.4_QTI, FW1.4_stab, FW1.4c_QTI, FW1.4c_stab, TNF1.4_stab, TNFP_QTI, TNFP_stab, YIELD1.4c_QTI, YIELD1.4c_stab, YIELD1.4nc_QTI, YIELD1.4nc_stab, YIELD1.4T_QTI, YIELD1.4T_stab; flowering and ripening precocity: FLOW; flowering and ripening precocity stability: RIP_QTI, RUN_stab; fruit quality: FEC, fec.a*, fec.b*, fec.C, Fec.H, Fec.L, GSH; fruit quality stability: FEC_QTI, Fec.b*_stab, Fec.C*_QTI, fec.C*_stab, fec.H_QTI, Fec.H_stab, Fec.L_QTI, Firm_QTI, GSH_stab; fruit shape: FAS, ITS, PUF, RCE; fruit shape stability: FAS_QTI, FAS_stab; physiological disorders: CCRACK, RCRACK; physiological disorders stability: BER_QTI, CCRACK_QTI, RCRACK_stab; Plant & inflorescence_architecture: FOL.

To reveal SNP–phenotype and SNP–stability associations underlying the genetic architecture of the traits and their stability, we tested (i) the SNP effects on the phenotypic mean of the trait across environments (meanQTL); (ii) stability assessed as the SNP effects modulated by the environment (QTI) (i.e. departure from stability); and (iii) the SNP effects on the stability index (stbQTL). We used the panel of 197 TCC accessions and 2946 markers with MAF >5% (Supplementary Table S8). Modern varieties are mostly hybrids (therefore, a heterosis effect may occur) and contain multiple wild resistance gene introgressions. In order to avoid the effect of this different genetic structure compared with traditional varieties, we excluded them to specifically focus on traditional variability. A total of 199 significant marker–trait associations (MTAs, *P*<10^-5^), consisting of 141 QTLs and involving 86 SNPs across 62 independent loci, were identified for the phenotypic means and the stability (Fig. 3; Supplementary Table S9). Out of them, 72 MTAs (47 QTLs covered by 58 SNPs in 29 loci) were associated with the phenotypic means (meanQTLs), 69 MTAs (46 SNPs in 35 loci) were in 53 QTIs, and 58 MTAs were in 41 stbQTLs (34 SNPs in 24 loci).

At the chromosomal level, the significant MTAs were distributed on all chromosomes (Fig. 3), except for chr10 and chr12, and with a preponderance on chr11 and chr01, with 45 and 37 MTAs, respectively (Supplementary Table S9). Additionally, QTLs were concentrated on different chromosomes depending on whether they were associated with meanQTLs, stbQTLs, or QTIs. QTL hotspots were found in chr01 and chr11 for meanQTLs, chr02 and chr05 for stbQTLs, and chr01 and chr08 for QTIs. Overall, we found loci where several QTLs co-localized (Fig. 3), which might be due to pleiotropic effects on multiple traits and/or stability, trait correlation, or linkage of different genes. The three main association hotspots mapped at chr11 (locus TCC_L62 with 18 QTLs and 43 MTAs), chr02 (locus TCC_L21 with 7 QTLs and 36 MTAs), and chr09 (locus TCC_L55 with 4 QTLs and 11 MTAs).

**Fig. 4.**
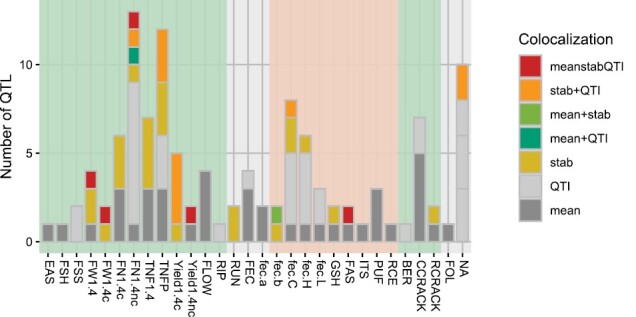
Number of meanQTLs, stbQTLs, and QTIs co-localizing at the same loci for each trait. Traits are represented on the *x*-axis and number of co-localizing or specific QTLs on the *y*-axis. The colour code represents the type of co-localization.

The comparison of the meanQTLs, stbQTLs, and QTIs for each trait at each locus revealed that the majority of QTLs were specific to either mean or stability (Fig. 4). Specifically, 34.19, 29.91, and 19.66% of QTLs were specific to meanQTLs, QTIs, and stbQTLs, respectively, while 5.98% of meanQTLs and stability QTLs (QTI, stbQTL, or both) and 10.26% of QTIs and stbQTLs co-localize at the same loci. All QTL classes co-localize exclusively in two loci for five traits, namely FN1.4nc at locus TCC_L04 in chr01, and FAS, FW1.4, FW1.4c, and Yield1.4nc at locus TCC_L62 (Supplementary Table S9).

### Assessment of the mode of inheritance of associated loci

**Fig. 5.**
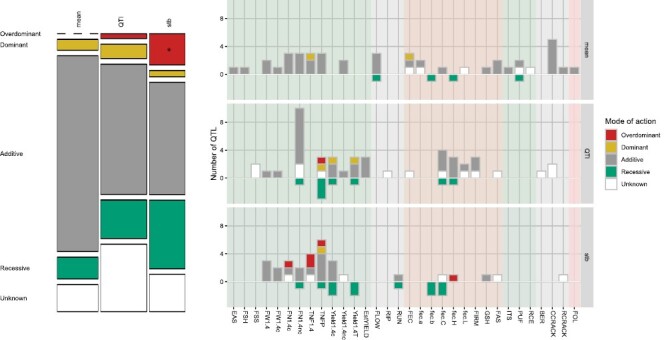
Mode of action of associated loci. (A) Mosaic plot representing the distribution of each type of mode of inheritance. Stars represent significance of enrichment at *P*-value >0.05. (B) Mode of inheritance by trait and type of QTL.

To further understand the genetic mechanisms of QTLs underlying the studied traits and their stability, and to classify these variants, we estimated the mode of inheritance (additive, dominant, recessive, and overdominant) for each SNP included within QTLs, and the direction of additive effects for the minor allele relative to the major allele, and the dominance deviation (Supplementary Table S10; Supplementary Figs S16, S17). We were able to assess the mode of inheritance and the direction of the effect for 116 QTLs (Fig. 5; Supplementary Table S10). Thirteen QTLs could not be analysed, either because they were QTIs for qualitative traits, or because one of the allelic states was represented by fewer than three accessions. For 12 more, the estimation of the mode of inheritance and the effect was not conclusive. The majority of QTLs showed an additive (57.45%, 81 QTLs, *P*<0.05) or recessive (16.31%, 23 QTLs) mode of inheritance (Fig. 5). When the distribution of mode of inheritance was compared across meanQTLs, stbQTLs, and QTIs (Fig. 5A; Supplementary Fig. S16), we found that stbQTLs had a significantly higher proportion of overdominant (12.20%) inheritance mode (*P*-value <0.05, *d*_ij_ >2), while no enrichment was found for the other QTL types. The traits showing non-additive inheritance modes mainly corresponded to those related to reproductive traits such as fruit number and yield, and also fruit colour CIELAB coordinate (Fig. 5B). Regarding the direction of the effect, in most QTLs (75.89%), the minor frequency allele increased the trait or its instability, with a similar distribution across all QTL types (Supplementary Table S10). A further examination of the 43 QTIs for the quantitative traits (Supplementary Figs S16, S17; Supplementary Table S10) indicated that 72.09% were detected in a single environment (El-Soda *et al.*, 2014), 18.60% showed changes in the magnitude of the allelic effect among environments but not in the direction, and ~9% presented cross-interacting allelic effects among environments.

### GWAS identified regions co-localizing with known QTLs

To further validate the efficiency of the TCC as a GWAS panel, we first compared the TCC associations with the meta-analysis of the entire TRADITOM collection (Pons *et al.*, 2022). Twenty-eight MTAs in 13 meanQTLs for 12 traits were detected in both collections (Supplementary Table S9). Of these, 12 MTAs in five QTLs were at the same SNP, two MTAs in one QTL at the same loci, and 13 MTAs in eight QTLs were associated with a related trait at the same SNP or loci (i.e. fruit colour CIELAB coordinates with FEC and ITS with fruit shape homogeneity traits). A larger number of QTLs were detected in the TRADITOM meta-analysis (Pons *et al.*, 2022), probably due to the differences in sample size (226 in TTC compared with 1315 in TRADITOM). The SNP–trait associations in TTC included mostly common SNPs (TCC GWAS was based on variants with MAFs ≥5%), while associations with very rare variants were not detected. TCC-GWAS may reflect mostly QTLs involved in the main phenotypic differences among traditional European varieties. The overlap between QTLs previously identified in the TRADITOM meta-analysis (Pons *et al.*, 2022) confirms that the TCC is a reliable collection for a GWAS panel. Regarding QTLs detected in previous works other than TRADITOM, 51 QTLs (36% of the total 141 QTLs) mapped to regions already associated with the trait or its stability (Fig. 3; Supplementary Table S9).

In total, 23 meanQTLs with 38 MTAs in 14 loci (chr01, chr02, chr05, chr06, chr08, and chr11) co-localized with known QTLs for the phenotypic mean. Furthermore, eight MTAs in five stbQTLs in four loci (chr01, chr08, and chr11), and 21 MTAs in 14 QTIs in 13 loci (chr01, chr08 and chr11) mapped in regions where GEI effects had been reported in previous studies (Supplementary Table S9).

The highest frequency of previously published QTLs mapped within the TCC_L62 locus on chr11 (~19.12%) (Supplementary Table S9). In the current work, the meanQTLs for FAS, FW1.4c, FW1.4, ITS and, RCE, the stbQTLs for FW1.4c and FW1.4, and the QTIs for FW1.4c and FW1.4 in TCC_L62, co-localized with known QTLs (see references in Supplementary Table S9). The region, harbouring *fas*/CLV3 (Xu *et al.*, 2015) and *fw11.3*/CSR (Mu *et al.*, 2017) (Fig. 3), two of the major genes regulating fruit weight and fasciation, has several pleiotropic effects on plants and reproductive organ architecture (Chu *et al.*, 2019; Pons *et al.*, 2022), and it has been also been identified as a plasticity regulatory hub (Diouf *et al.*, 2020) for fruit weight and other traits. The next locus that included previously published QTLs was TCC_L21 (Fig. 3; Supplementary Table S9), with ~10.29% of total published QTLs. In this locus, we detected multiple meanQTLs and stbQTLs for fruit colour traits. These QTLs overlap with a previously identified QTL for external fruit colour (Pons *et al.*, 2022), and with the neoxanthin synthase NXS2/ABA4 gene (Solyc02g063170), which catalyses the last step of the carotene biosynthesis pathway and the first step of the abscisic acid (ABA) biosynthesis pathway (North *et al.*, 2007; Neuman *et al.*, 2014). Other published QTLs (Supplementary Table S9) also included the cutin deficient 2 gene (*cd2*) for cracking (Isaacson *et al.*, 2009), WUS/lc for fasciation (Muños *et al.*, 2011), and QTLs for which the causal gene remains to be determined, such as flw5.1 and flw8.1 (Diouf *et al.*, 2020) (flowering time), ec_a*2.1 (external fruit colour) (Barrantes *et al.*, 2016), fw3.1 (fruit weight) (Barrantes *et al.*, 2016), bpi8.1 (puffiness) (Van Der Knaap and Tanksley, 2003), frn1.1 (fruit number) (Gonzalo *et al.*, 2022), NFr1.1 salinity (specific number of flowers in salinity) (Diouf *et al.*, 2018), ppfset1.2 (plasticity of fruit set) (Diouf *et al.*, 2020), and pfrn2.1_T31_2E (plasticity of fruit number at different temperatures) (Gonzalo *et al.*, 2022).

### Novel QTLs and candidate genes

We explored genomic regions that flanked (<2 Mb) meanQTLs, stbQTLs, and QTIs to scan for candidate genes that may be physically proximal to associated SNPs. Within 141 QTL regions, we identified a total of 11 094 annotated genes, ranging from 28 to 550 genes per QTL, with a mean of 259 genes per QTL. To select candidate genes within the QTL region, in the case of meanQTLs, we prioritized genes with reported roles controlling the trait, while genes with reported roles in the integration of both external (biotic and abiotic stress) and internal factors (e.g. phytohormones) were prioritized for stbQTLs and QTIs (Fig. 3; Supplementary Table S9). We searched in the literature and publicly available databases for both tomato genes and tomato orthologues (noted here as ‘like’), having a demonstrated role in the studied traits or their stability. Many of the novel QTLs included candidate genes whose natural variation had not been associated in tomato with the trait, but their implication in the regulation of the trait had been demonstrated through mutant analyses (Fig. 5; Supplementary Table S9). For instance, CCRACK meanQTLs in the TCC_L46 locus contained Solyc06g082980, a 3-β-hydroxysteroid dehydrogenase/C-4 decarboxylase (3-betaHSD/D) involved in a hypercracking tomato fruit phenotype (Schrick *et al*., 2012). Fruit colour (FEC, fec.a*, and fec.b*) mean QTLs in loci TCC_L03, TCC_L23, TCC_L43, TCC_L55, and TCC_L60 included genes regulating the concentration of carotenoids in fruits such as *sit* (Galpaz *et al.*, 2008), SlEIN2 (Gao *et al.*, 2016), *LeETR4* (Tieman *et al.*, 2000), or TAG1 (Vrebalov *et al.*, 2009). The FW1.4 meanQTL in the TCC_26 locus contained SlGA20ox1 (Solyc03g006880), which plays an important role in controlling fruit weight (Chen *et al.*, 2016). The GSH meanQTL in locus TCC_L61 included SlARF10A (Solyc11g069500), a transcriptional activator controlling chlorophyll accumulation and the expression of SlGLK2 (Yuan *et al.*, 2018), the gene responsible for the uniform ripening (*u*) mutation affecting chloroplast biogenesis in fruit and the intensity of the green shoulder (Powell *et al.*, 2012). Easiness of fruit to detach from the pedicel (EAS) meanQTLs in locus TCC_L62 harboured the transcription factor genes SlREV (Solyc11g069470) and LBD1 (Solyc11g072470) involved in the development of the abscission zones of the flower pedicel (Hu *et al.*, 2014) and the leaf (Sundaresan *et al.*, 2016), respectively. The PUF meanQTL in the TCC_L53 locus contained SICDKA1 (Solyc08g066330), whose mutation diminished the amount of jelly in the placenta (Czerednik *et al.*, 2015).

Furthermore, we found within or near stbQTLs and QTIs (Fig. 5; Supplementary Table S9, and references therein), tomato genes or genes whose orthologues had a demonstrated role in abiotic stress response (ALA3-like, SlJUB1, SlPDI6-1, RBOH-C/E, DRIP2-like, dnaJ, SlMYB12, SlDHAR4, MSL, SLNAC042, 5pt2, KIN1-like, and SlMAPKKK2), biotic response (I2, DCL2-like, RFC3-like, WRKY37, and SlmSOL2), auxin biosynthesis and signalling (SlTAR3 and SlARF7B/SlARF19B), ABA (RDUF2-like, AIP2, ABI8-like, SLY2-like, and NXS2/ABA4), ethylene signalling (SlEIL5, SlERF1-5, and FUL1/TDR4 SlEIN2), gibberellin signalling (GH3-like and SlGRAS3), brassinosteroid signalling (BIN4-like and *cu-3*/SlBRI1), polyamine biosynthesis and transport (SLOAT, SlLAT3, and SPM), cytokinin biosynthesis (SlIPT4 and SlCKX1), and jasmonate biosynthesis and signalling (SlLOX8 and SlJAZ9). Some of these genes were also reported in hormone–stress crosstalk. Among these genes in stbQTLs and QTIs, some of them had been previously associated with stability roles in tomato or other plants (Supplementary Table S9). For instance, the FN1.4nc QTI in TCC_L04 harboured Solyc01g011100, the tomato orthologue of the *Arabidopsis thaliana* phospholipid-transporting ATPase (AtALA3), a gene affecting adaptability of rosette size and fecundity in response to heat stress (McDowell *et al.*, 2013). The TNFP stbQTL in TCC_L41 included the auxin response factor SlARF7B/SlARF19B (Solyc05g047460), which stabilizes the developmental fruit shape variability (de Jong *et al.*, 2011; Israeli *et al.*, 2019). The FN1.4c QTI and TNF1.4 stbQTL in TCC_L30 included the transcription factor Blade-On-Petiole BOPa/SlBOP1 (Solyc04g040220), which canalizes the leaf morphospace (Ichihashi *et al.*, 2014). Moreover, for some candidate genes in stbQTLs and QTIs, the previously reported mutation effect on the phenotype was obvious only in one specific environment (Supplementary Table S9). Among them were the NXS2/ABA4 (Solyc02g063170) (North *et al.*, 2007; Neuman *et al.*, 2014) in fruit colour stbQTLs in TCC_L21, the class III homeodomain leucine-zipper transcription factor CNA/pf1/SlHB15A (Solyc03g120910) (Clepet *et al.*, 2021) in the FN1.4c stbQTL, the TNF1.4 QTI in TCC_L29, and the isopentenyltransferase SlIPT4 (Solyc09g064910) (Žižková *et al.*, 2015) in the TNFP QTI in TCC_L58.

### Haplotype analysis of targeted loci in the TCC and TRADITOM collections

Finally, we assessed the ability of the TCC to capture haplotype diversity within the entire TRADITOM collection. We selected two loci associated with FW or PUF in both collections: TCC_62 locus (named L197 by Pons *et al.* (2022) on chr11 associated with FW, and the TCC_L53 locus (named L160 by Pons *et al.* (2022) on chr08 associated with PUF (Supplementary Table S9). The TCC genotypic data were utilized to identify the minimum set of SNPs that define haplotype blocks at each locus in the TCC and to determine haplotypes across the entire TRADITOM collection. In both collections, TCC_L62 exhibited three haplotype blocks (HA, HB, and HC), consisting of eight SNPs and 21 distinct haplotypes in HA, five SNPs and 12 haplotypes in HB, and three SNPs and eight haplotypes in HC. The TCC_L53 locus displayed a single haplotype block (L53 block) with three SNPs and eight different haplotypes (Supplementary Table S11; Supplementary Fig. S18).

Among TCC_L62 and TCC_L53 haplotypes, seven and two, respectively, occurred at a frequency >10% (major haplotypes), while 12 and five were at a frequency <10% in each case (minor haplotypes). The remaining haplotypes, with frequencies <1%, were considered rare haplotypes (Supplementary Table S11; Supplementary Fig. S18). Similar haplotype frequencies were observed between the TCC and the entire TRADITOM collection (Supplementary Table S11; Supplementary Fig. S18), with only slight frequency increases for a few of them in the TCC. Some rare haplotypes could not be identified in either collection (Supplementary Table S11; Supplementary Fig. S18).

**Fig. 6.**
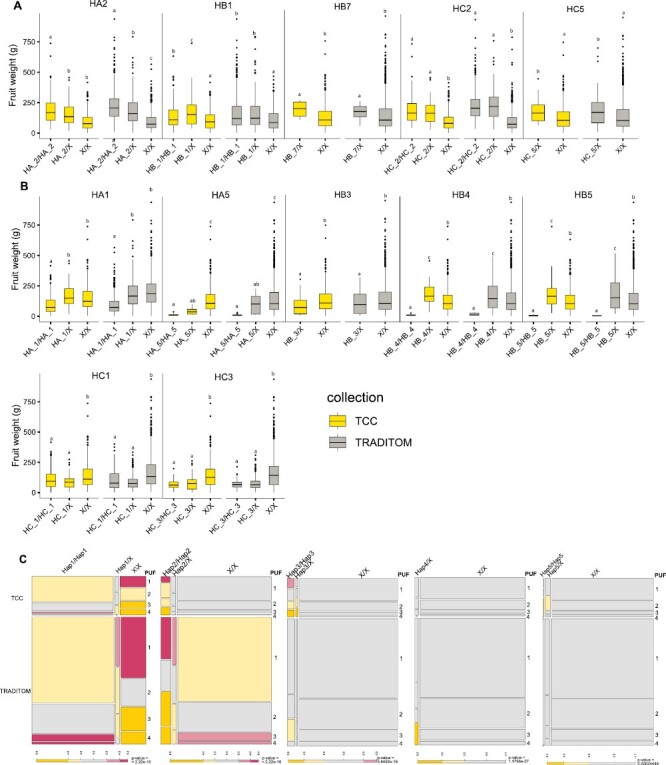
Association between absence (0 copies) and presence (1 or 2 copies) of haplotypes in blocks of TCC_L62 and TCC_L53 loci and FW and PUF in the TCC and TRADITOM collection. Absence of target haplotype is coded by ‘X’ haplotype. Only haplotypes significantly associated with the traits (*P*<0.05) are shown. (A and B) Boxplots show the effects of TCC_L62 haplotypes on FW in the TCC and TRADITOM collection. Each panel corresponds to one haplotype. (A) Haplotypes increasing FW. (B) Haplotypes decreasing FW. Different letters above the box plots indicate statistically significant differences (*P*<0.05). The *x*-axis depicts the haplotype and the *y*-axis the FW. The horizontal bar inside the box plot indicates the mean value. Bars in the box plot represent the SD, and dots correspond to outliers. (C) Mosaic plots showing the effect of the presence/absence for TCC_L53 PUF-associated haplotypes (*P*<0.05). The colour of the mosaic depicts the enrichment, evaluated by departure of Pearson residuals (*d*_ij_) from the expected value. Residuals with |*d*_ij_|>4 have an approximate *P*-value <0.001 and with |*d*_ij_|>2 have an approximate *P*-value <0.05. Yellow colour indicates over-representation and red under-representation.

For each block in each locus, we also analysed the effect of haplotypes on the target trait in both collections. Haplotype effect was highly significant for all blocks, with a low or non-significant interaction effect between haplotype and collection effect in most cases (Supplementary Table S12). For the TCC_L62 locus, HA2, HB1, HB7, HC2, and HC5 haplotypes significantly increased (*P*<0.05) FW (Fig. 6A; Supplementary Figs S19–S21), while HA1, HA5, HB3, HB4, HB5, HC1, and HC3 haplotypes significantly decreased it (Fig. 6B; Supplementary Figs S19–S21). With a few exceptions, the haplotype effect was the same between TCC and the entire core collection (Fig. 6B; Supplementary Figs S19–S21). Regarding TCC_L53, Hap1 was significantly associated with no fruit puffiness, and Hap2, Hap3, Hap4, and Hap5 were related to intermediate and severe fruit puffiness (Fig. 6C; Supplementary Fig. S22). The significant haplotype×collection interaction (Supplementary Table S12) was due to a change of the magnitude of the estimated allelic effects among collections, although the main effects were significant in both collections. The rest of the haplotypes appear to be neutral with no effect on the phenotype (Supplementary Figs S19–S22).

## Discussion

The objective of the present work is to estimate and identify the genetic architecture of agro-morphological traits and its stability in European traditional tomatoes, taking advantage of the availability of the TRADITOM collection (Blanca *et al.*, 2022; Pons *et al.*, 2022). However, the size of this collection restricts its application in research, especially for METs. To overcome this issue, we have generated the TCC European traditional multipurpose core collection consisting of 226 European traditional tomato varieties (Supplementary Table S3) that fulfilled the initial size requirement of 5% and 20% of the original collection for an ideal core collection (Brown and Spillane, 1999). Core collections are used as a reference set of accessions for the whole collection, enabling more efficient and effective characterization and management (van Heerwaarden *et al.*, 2013). Given that the genetic diversity within the tomato accessions is limited (Blanca *et al.*, 2022) and the GBS technique used for the genomic characterization of the TRADITOM collection cannot capture all genetic diversity, we used a mixed approach (genotypic, phenotypic, and geographical) to select the TCC. This method would ensure that (epi)genetic variation, missed by the initial genotyping, was included in the core collection. Furthermore, the collection was supplemented with accessions of historical relevance (popularity, prestige, or role in breeding history). Although this process might not maximize genetic diversity, it has been demonstrated that core collections designed by mixing genetic information with expert knowledge (popularity, prestige, role in breeding history, or presence of phenotypic features of interest) provided a similar efficiency in optimizing the retention of genetic diversity (Urrestarazu *et al.*, 2019). Accordingly, the genetic, phenotypic, and geographical diversity between the TCC and the TRADITOM collection were equivalent (Fig. 1; Supplementary Figs S2–S5). Further, the TCC satisfied nucleotide diversity, structure, and co-ancestry requirements to be used as a GWAS panel (Supplementary Figs S4, S6) and validated previously reported QTLs (Supplementary Table S9). Moreover, using two loci as examples, one associated with a quantitative trait (TCC_L62) and the other with a qualitative trait (TCC_L53), we demonstrated that both the frequencies of the most common haplotypes and haplotype–trait associations were similar between the TCC and the TRADITOM collections (Fig. 6; Supplementary Figs S18–S22; Supplementary Tables S11, S12). These results indicate that TCC, despite being based on a small number of accessions, successfully captured the most common haplotypes of the TRADITOM. However, most importantly, TCC provides both a valuable germplasm resource for the identification of novel QTLs and an efficient way to find beneficial haplotypes without the need for extensive phenotyping.

MET analysis of TCC accessions (most of them adapted to specific geographical areas; Casañas *et al.*, 2017) has enabled us to study their response to the environment, and to identify new as well as previously detected QTLs (Figs 2–4; Supplementary Tables S5–S9). Sixty-two loci involved in plant and fruit traits and 33 loci affecting the trait stability were detected (Fig. 3; Supplementary Table S9). We also proposed candidate genes within the QTL regions that could be involved in the studied traits and their stability (Fig. 3; Supplementary Table S9). Candidate genes for stbQTLs and QTIs had molecular functions involved in stress and hormone signalling, while developmental genes were more often associated with meanQTLs (Fig. 3; Supplementary Table S9). Many studies have suggested that hormonal systems and network internal developmental signals and processes integrate environmental cues and are involved in the regulation of trait plasticity (Weng *et al.*, 2016; Kong *et al.*, 2017; Xiao *et al.*, 2017; Qiao and Stepanova, 2021; Jia *et al.*, 2022). Therefore, these candidate genes provide targets for functional analysis to better characterize the complex polygenic regulation of the agro-morphological traits and their stability.

We phenotyped and estimated the stability of TCC accessions (Supplementary Table S3) together with modern varieties and inbred lines (Supplementary Table S4) to position the traditional tomatoes within the phenotypic diversity landscape of the tomatoes that are currently cultivated in Southern Europe (Fig. 2; Supplementary Figs S7–S14; Supplementary Tables S5–S7). In general, traditional tomatoes were more similar to the modern than to the traditionalized tomatoes (Supplementary Figs S7–S14). This could be due, in part, to traditionalized tomatoes being mostly medium sized, non-fasciated round processing tomatoes (Pons *et al.*, 2022), while modern tomatoes have the same typologies as traditional ones. Indeed, traditional tomatoes mainly differed from traditionalized (with the exception of fruit size and shape parameters) and from modern tomatoes in earliness, yield, FSS, FSH, FIRM, EAS, and resistance to BER and cracking traits (Supplementary Figs S7–S14), which have for long a time been targets for modern tomato breeding (Causse *et al.*, 2020). Our results also indicated that although modern varieties generally showed better values for those traits, several traditional varieties showed similar or even better performance in yield and other agro-morphological traits than modern ones (Supplementary Figs S7–S14). This indicates that, contrary to popular belief (Casañas *et al.*, 2017), some traditional varieties exhibit excellent agronomic performance, even as good as or better than the modern counterparts. Further, the large GEI found (Fig. 2; Supplementary Fig. S13; Supplementary Table S6) suggests that selection for adaptation to specific environments should be necessary to maximize some traits such as yield.

With the exception of a few traits, there was no general trend for higher stability in either modern or traditionalized tomatoes compared with traditional tomatoes (Fig. 2; Supplementary Figs S7–S14; Supplementary Table S7). Differences among the tomato groups mostly reflect particular and distinctive trait characteristics of each tomato group. Therefore, the stability of agro-morphological traits had undergone a convergent selection independently by both farmers and modern breeders. The similar trait stability and plasticity among tomato groups probably indicates an ancestrally selected attribute that favours reproductive success and maximizes higher plant adaptation in nature and in agricultural fields (Fisher *et al.*, 2017). In addition, as evidenced by the significant correlations (Supplementary Fig. S15), stability and trait values are not completely independent, and increasing trait values could result in a trade-off in stability. A significant correlation between phenotypic mean and stability might be the result of the action of pleiotropic genes (Diouf *et al.*, 2020). Our results indicated that both common and specific loci controlled the traits and stability variation in traditional tomato (Fig. 4; Supplementary Table S9). However, the proportion of mean and stability shared loci was only 5.98%. A possible explanation for the discrepancy between the small percentage of shared loci and the magnitude of observed correlations is that, most probably, shared loci are those with major effects on the phenotype [such as *fas*/CLV3 (Xu *et al.*, 2015) and *fw11.3*/CSR (Mu *et al.*, 2017) in TCC_L62] that have gone through extensive directional selection towards the allele that maximizes the trait, which in turn favoured the increase in plasticity (Springate *et al.*, 2011; Melo and Marroig, 2015). The large proportion of independent mean and stability loci in European traditional tomato germplasm (Fig. 4; Supplementary Table S9) indicated that, as reported previously in other tomato populations (Alseekh *et al.*, 2017; Diouf *et al.*, 2020), the stability gene regulatory model is the predominant one. This would facilitate tomato adaptation to and flexibility in different environments (Springate *et al.*, 2011).

A noteworthy consequence of this is that specific stability loci could be targets for breeding without affecting the phenotypic mean, or vice versa. Since the mode of inheritance of a trait does not affect its stable or plastic nature (Fisher *et al.*, 2017), understanding the inheritance mechanism and the direction of the effect of identified QTLs would provide valuable and practical information for planning breeding strategies that optimize allele combinations of favourable traits or their stability. We found QTLs with additive, recessive, dominant, and overdominant inheritance (Fig. 5; Supplementary Figs S16, S17; Supplementary Table S10). From a global view, most of the QTLs identified displayed an additive inheritance model, with the exception of stbQTLs, which were enriched in overdominant QTLs. We dealt with genotyping data from pools of plants of traditional accessions (Blanca *et al.*, 2022; Pons *et al.*, 2022), which may have inherent intra-varietal heterogeneity (Casañas *et al.*, 2017). Heterozygous loci indicate genetic heterogeneity, which could have segregated in our experimental set up. Therefore, the number of strictly additive loci may be overestimated, while those with dominance effects may be underestimated. Further experiments and crosses are needed to better estimate the inheritance mode.

In summary, we have demonstrated that TCC is an efficient genetic tool for investigating the genetic diversity of traditional tomato germplasm conserved in the Southern European genebanks. The relatively small size of the TCC allows complex biological questions to be addressed with a reasonable effort. For example, the previous genomic analysis of the entire TRADITOM collection (Blanca *et al.* 2022) was based on GBS markers. Consequently, large regions of the genome were not analysed, and complex genomic features such as structural variation and epigenetics could not be addressed. Resequencing the TTC would provide valuable insights into these features. Furthermore, the TCC has proven useful in dissecting complex traits, including environmental interactions and trait stability. This opens the door to utilizing the collection for investigating molecular traits such as transcriptome, proteome, and metabolome profiles, as well as their response to different stresses. The TCC will enhance the use of traditional tomato accessions, making this germplasm accessible for breeders and contributing to develop stable or locally adapted improved varieties.

## Supplementary data

The following supplementary data are available at *JXB* online.

Fig. S1. PCoA of 1850 traditional accessions and varieties from five different collections.

Fig. S2. Original collection sites of the traditional European tomato accessions.

Fig. S3. SNP distribution in the TRADITOM and TCC collections.

Fig. S4. Comparison of nucleotide diversity between TRADITOM and the TCC collections.

Fig. S5. Comparison of quantitative trait frequency distribution in the TCC and the TRADITOM collections.

Fig. S6. Population structure and kinship.

Fig. S7. Boxplot showing the differences in the genotypic mean and the stability of traits related to fruit weight and fruit number among the three tomato groups.

Fig. S8. Boxplot showing the differences in the genotypic mean and the stability of traits related to fruit yield among the three tomato groups.

Fig. S9. Boxplot showing the differences among the three tomato groups in the genotypic mean and the stability of easiness to detach from the pedicel, foliage density, fasciation, fruit set sequence, fruit size homogeneity, and green shoulder.

Fig. S10. Boxplot showing the differences in the genotypic mean and the stability of traits related to flowering and ripening precocity.

Fig. S11. Boxplot showing the differences in the genotypic mean and the stability of traits related to fruit quality among the three tomato groups.

Fig. S12. Boxplot showing the differences among the three tomato groups in the genotypic mean and the stability of irregular transversal section, foliage density, and physiological disorders.

Fig. S13. Interaction plots of trait means for each accession across locations.

Fig. S14. Mean performance versus stability GGE genotype-focused biplots for accession and location.

Fig. S15. Pearson’s correlations across the different locations.

Fig. S16. Mosaic plots showing the distribution of each mode of inheritance among the three QTL classes (meanQTL, stbQTL, and QTI).

Fig. S17. Location-specific allelic effects of MTAs in identified QTIs.

Fig. S18. Linkage disequilibrium (LD) and haplotype analysis of TCC_L62 and TTC_L53 loci in the TCC and TRADITOM collection.

Figu. S19. Association between absence and presence of TCC_L62 HA haplotypes on FW in the TCC and TRADITOM collection.

Fig. S20. Association between absence and presence of TCC_L62 HB haplotypes on FW in the TCC and TRADITOM collection.

Fig. S21. Association between absence and presence of TCC_L62 HC haplotypes on FW in the TCC and TRADITOM collection.

Fig. S22. Association between absence and presence of TCC_L53 locus hap6 haplotype on PUF in the TCC and TRADITOM collection.

Table S1. Field cultivation conditions for each location.

Table S2. Traits studied and respective abbreviations.

Table S3. Passport data for accessions included in this work.

Table S4. Modern counterparts and their characteristics.

Table S5. Phenotypic mean values per accession.

Table S6. Analysis of phenotypic variance and heritability.

Table S7. Estimated stability indexes.

Table S8. Genotypic data.

Table S9. Summary of GWAS and GWEIS results.

Table S10. Mode of inheritance of QTLs.

Table S11. Haplotypes and haplotype frequency in target loci in the TCC and TRADITOM collection.

Table S12. ANOVA of haplotype and collection effects on trait value.

erad306_suppl_Supplementary_Protocols_S1-S2_figures_S1-S22

erad306_suppl_Supplementary_tables_S1-S12

## Data Availability

The data supporting the findings of this study are available within the paper and within its supplementary data published online. GBS data raw are available in the SRA (https://www.ncbi.nlm.nih.gov/sra) under accession numbers PRJNA722111 and PRJNA774172. Datasets used to evaluate the core collection with respect to the entire TRADITOM collection are available in Pons *et al.* (2022).

## Abbreviations

AEC: average environment coordination
BLUP: best linear unbiased predictor
BLUPg: best linear unbiased prediction for genotype effect
BLUPge: best linear unbiased estimation for genotype–environment interaction
CCRACK: presence and incidence of concentric cracking
EAS: easiness of fruit to detach from the pedicel
EstYIELD: total estimated yield per plant (kg per plant)
FAS: fruit fasciation
FEC: fruit external colour
fec.a*: CIELAB fruit external colour coordinate a
fec.b*: CIELAB fruit external colour coordinate b
fec.C*: CIELAB fruit external colour chroma
fec.H: CIELAB fruit external colour hue angle
fec.L*: CIELAB fruit external colour coordinate L
FIRM: fruit firmness
FLOW: flowering earliness
FN1.4c: number of commercial fruits on trusses 1–4
FN1.4nc: number of non-commercial fruits on trusses 1–4
FOL: foliage density
FPS: fruit predominant shape
FSH: fruit size homogeneity
FSS: fruit set sequence
FW1.4: mean fruit weight (g) on trusses 1–4
FW1.4c: mean of commercial fruit weight (g) trusses 1–4; FW1.4nc, Mean of non-commercial fruit weight (g) trusses 1st to 4
G×E: genotype by environment
GBS: genotyping by sequencing
GEI: genotype by environment interaction
GGE model: genotype main effects GEI model
GH: growth habit
GSH: green shoulder
GWAS: genome-wide association study
H^2^: broad sense heritability
IPCA: interaction principal components axis
ITS: irregular transversal section
LD: linkage disequilibrium
MAF: minimum allele frequency
MDS: multi-dimensional scaling
meanQTL: SNP effects on the phenotypic mean of the trait across environments
MET: multi-environment trial
MTA: marker–trait association
NID: normally, identically and independently distributed
PCoA: principal coordinate analyses
PUF: puffiness appearance
QTI: QTL by environment interactions
QTL: quantitative trait locus
*r* ^2^: inter-variant allele counts squared correlations
RCE: ribbing at calyx end
RCRACK: presence and incidence or radial cracking
RIP: ripening earliness
RUN: ripening uniformity of the whole plot
SNP: single nucleotide polymorphism
stbQTL: SNP effects on the stability index
TCC: traditional tomato core collection
TNF1.4: total number of fruits on trusses 1–4
TNFP: total number of fruits per plant
WAASB: weighted average of absolute scores
Yield1.4c: commercial yield on trusses 1–4 (kg per plant)
Yield1.4nc: non-commercial yield on trusses 1–4 (kg per plant)
YIELD1.4T: total yield on trusses 1–4 (kg per plant)
