# Supplementary material for: Diversity and genetic architecture of agro-morphological traits in a core collection of European traditional tomato
Source: J Exp Bot. 2023 Aug 1;74(18):5896–916. doi: 10.1093/jxb/erad306 (PMC10540738; doi:10.1093/jxb/erad306)
Supplement: erad306_suppl_Supplementary_Protocols_S1-S2_figures_S1-S22 [file erad306_suppl_supplementary_protocols_s1-s2_figures_s1-s22.pdf]

Supplementary Information for

Diversity and genetic architecture of agro-morphological traits in a Core Collection of European traditional tomato

Clara Pons[1,2¥], Joan Casals[3¥], Matthijs Brower[4¥], Adriana Sacco[5], Alessandro Riccini[6], Patrick Hendrickx[4], Maria del Rosario Figás[1], Josef Fisher[7], Silvana Grandillo[5], Andrea Mazzucato[6], Salvador Soler[1], Dani Zamir[7], Mathilde Causse[8], Maria José Díez[1], Richard Finkers[4], Jaime Prohens[1], Antonio Jose Monforte*[2], Antonio Granell*[2]

* Antonio Jose Monforte.

Email: amonforte@ibmcp.upv.es

* Antonio Granell.

Email: agranell@ibmcp.upv.es

This PDF file includes:

Supplementary Protocols S1-S2

Supplementary Figures S1 to S22

Supplementary Protocol S1. Development of a multipurpose Core Collection in European traditional tomato

To create a core collection of European traditional tomato representative of 15-20% of the initial accessions (Brown and Spillane, 1999) in TRADITOM collection, we used a random subset of 16077 markers and 1784 accessions (1342 European traditional tomatoes plus 439 accessions from other origins, and representing the full range of expected genetic variation within *S. lycopersicum* around the world (see Material and Methods) (Causse *et al.*, 2013; Aflitos *et al.*, 2014; Lin *et al.*, 2014; Pons *et al.*, 2022; Blanca *et al.*, 2022) and phenotypic data for 67 traits from 1489 accessions (Pons *et al.*, 2022). The 439 extra accessions were used to put into context the genetic diversity within the global European traditional pool compared with the overall tomato diversity (Supplementary Fig. S1). The core collection was selected using a mixed approach. First, within the traditional accessions, two sub-core sets of entries (accessions in the core collection) were selected based on genotypic and phenotypic diversity. The first sub-core set consisted of 57 accessions selected to optimize genetic diversity within the first two PCoA axis calculated with the SNP matrix. The second sub-core set consisted in of 133 entries representing diversity in quantitative and qualitative phenotypic traits.

Subsequently, we optimized the core by assessing that all geographical regions present in the original collection were represented (Supplementary Fig. S2A). In total, 39 entries were selected to fill geographical gaps based on passport data. Then, we merged the three subsets of entries coming from the three previous approaches, resulting in a total of 217 non-redundant traditional entries. Thirty-five entries, for which no seeds were available or that segregated in the initial field trials, were replaced by accessions with historical relevance and tightly clustered to the selected entry in the PCoA biplot. The final European traditional tomato core collection (TCC; Supplementary Table S1) was composed of 226 entries (16.7% of the original collection). The TCC is composed by 190 entries representative of the genotypic, phenotypic and geographical diversity cultivated in the Mediterranean basin between 1950 and 2015 (Pons *et al.*, 2022; Blanca *et al.*, 2022), supplemented with 35 accessions with historical relevance. Following Blanca *et al.* (2022), 121 accessions of the TCC were classified as true vintage/landraces , 67 as “traditionalized” (varieties classified as traditional according with their passport but they were developed by local framers from obsolete commercial varieties that contained resistance gene introgressions) and the remaining 38 were unclassified (Supplementary Table S2)

Supplementary Protocol S2. Evaluation of the core collection

The representativeness of the 226 entries of the TCC relative to the original TRADITOM collection was assessed by comparing the diversity captured in the TCC compared with the whole TRADITOM based on passport, genotyping and phenotyping datasets previously generated by Pons *et al.* (2022). MDS based on genotypic data (110,909 markers) was conducted to assess whether the spatial distribution of the TCC represented the genetic diversity of the entire TRADITOM (Fig. 1A). Both collections showed similar distribution of accessions in the MDS space and the TCC covered the genetic variation of the entire TRADITOM (Fig. 1A). Additionally, we compared the percentage of SNP polymorphism, SNP distribution, MAF, nucleotide diversity (π) among the two collections. The average nucleotide diversity in TRADITOM was π = 0.0067 (5,715 SNPs (5.15 %) with MAF ≥0.01 and 4,146 and 457 SNPs having MAF ranging from 0.01 to 0.050 and 0.05 to 0.1, respectively, Fig. 1B and Supplementary Fig. S3A). SNPs showing MAF ≥0.01 were evenly distributed across the 12 chromosomes (Supplementary Fig. S3B), with an average of 439 SNPs per chromosome, ranging from 143 to 832 on chr7 and chr9, respectively. All these parameters were, in general, comparable between the entire TRADITOM and the TCC

collections (Fig. 1B, Supplementary Fig. S3 and Supplementary Fig. S4). However, TCC showed a π gain of 36.3% (π = 0.0095, p-value < 2.2e-16) respect to TRADITOM, especially in chr04, chr05, chr11 and chr12 (Supplementary Fig. S4B). The frequency of some rare alleles in the entire TRADITOM increased in the TCC (p-value = 3.7e-6; Fig. 1B), which is also reflected in SNP density along chromosomes (Supplementary Fig. S3A). In the TCC, 9,073 (8.18%) SNPs showed MAF≥0.01, of them 6,217 and 1,247 SNPs with MAF ranging from 0.01 to 0.050 and 0.05 to 0.1, respectively (Fig. 1B and Supplementary Fig. S3A).

The geographical distribution of core collection was further analysed, being similar between TCC and the entire TRADITOM (Fig.1C, Supplementary Fig. S2B). Regarding to phenotypic variation, 15 traits (nine quantitative and six qualitative) related to plant architecture, fruit morphology and fruit quality were studied (Fig. 1D and Supplementary Fig. S5). Violin plots showed that in general the means and range of variation for the nine selected quantitative traits were similar among collections (p-value <0.01), with the exception of *FIRM* (Fig. 1D). In the case of qualitative traits (Supplementary Fig. S5), TCC covered all the ranges of trait categories represented in the TRADITOM collection, however frequency distribution indicated non-homogeneity of distribution among the entire TRADITOM and TCC (Supplementary Fig. S5)

We assessed the suitability of TCC collection as GWAS panel. A GWAS panel should contain nucleotide diversity equivalent to that of a larger panel, as well as low population structure and low kinship among its members to avoid spurious marker–trait associations (Kumar *et al.*, 2020). The TCC increased nucleotide diversity compared with the entire TRADITOM (Supplementary Fig. S4). Population structure analysed by MDS (Supplementary Fig. S6A) showed a compact cluster that includes a high proportion of entries and some additional scattered small groups that include traditionalized entries. The kinship coefficients matrix analysis confirmed these small groups (Supplementary Fig. S4B), but indicated that 93% of pairwise kinship coefficients were lower than 0.25% (Supplementary Fig. S4C), indicating low to moderate level of genetic relatedness among most of the TCC entries. The TCC, therefore, preserved the majority of variation of the entire TRADITOM and fulfilled the requirements for a collection to be used as a GWAS panel.

References

Aflitos S, Schijlen E, De Jong H, *et al.* 2014. Exploring genetic variation in the tomato (Solanum section Lycopersicon) clade by whole-genome sequencing. Plant Journal **80**, 136–148.

Blanca J, Pons C, Montero-Pau J, *et al.* 2022. European traditional tomatoes galore: a result of farmers' selection of a few diversity-rich loci. Journal of Experimental Botany **73**, 3431–3445.

Causse M, Desplat N, Pascual L, *et al.* 2013. Whole genome resequencing in tomato reveals variation associated with introgression and breeding events. BMC Genomics **14**, 791.

Lin T, Zhu G, Zhang J, *et al.* 2014. Genomic analyses provide insights into the history of tomato breeding. Nature Genetics **46**, 1220–1226.

Pons C, Casals J, Palombieri S, *et al.* 2022. Atlas of phenotypic, genotypic and geographical diversity present in the European traditional tomato. Horticulture Research **9**, uhac112.

Spillane C. 1989. Implementing core collections - principles , procedures , progress , problems and promise - by A . H . D . Brown and. In: Johnson RC,, In: Hodgkin T, eds. Core collections for today and tomorrow. Rome:IPFRI, 7–17.

Supplementary figures

A

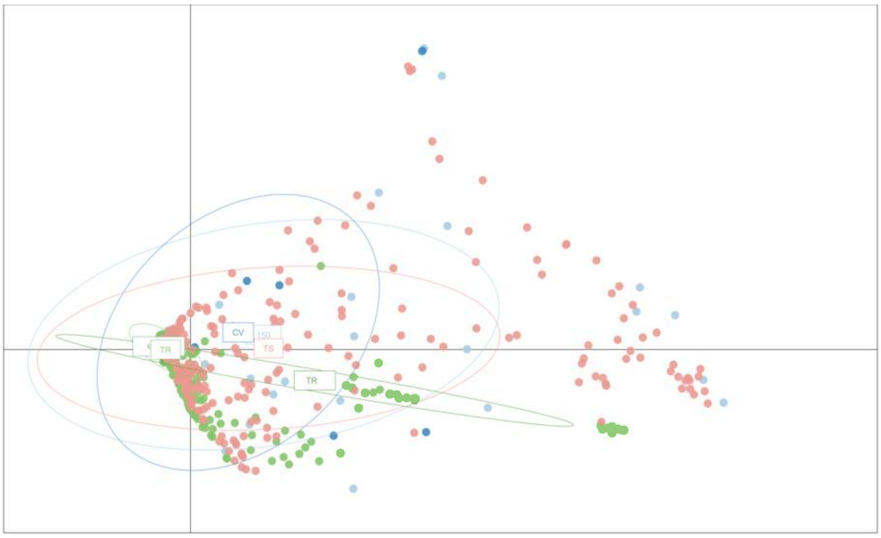

B

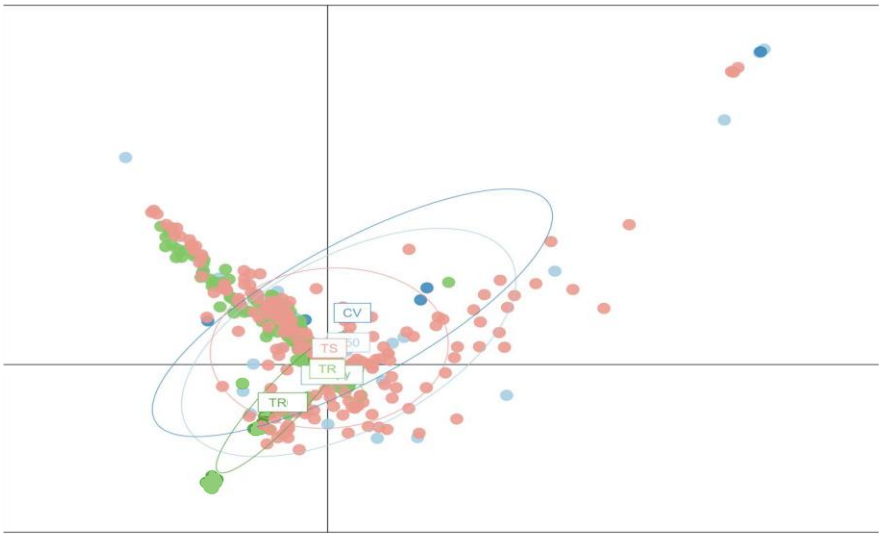

Supplementary Figure S1. PCoA analysis of 1850 traditional accessions and varieties from five different collections. The collections are: accessions from the TRADITOM project (TR, green), MAGIC parents (CV, dark blue), re-sequenced (150, light blue), and 350 re-sequenced (TS, pink). The data from 16077 polymorphic SNP markers were used for the analysis. Collections are shown within their 95% inertia ellipses. (A) Biplot of the first two axes. PC1 (x-axis) explains 33.4% of the variation, PC2 (y-axis) 4.18%. (B) Biplot of the second and third axes. The second axis (x-axis) explains 4.2% of the variation, the third axis (y-axis) 2.2.%.

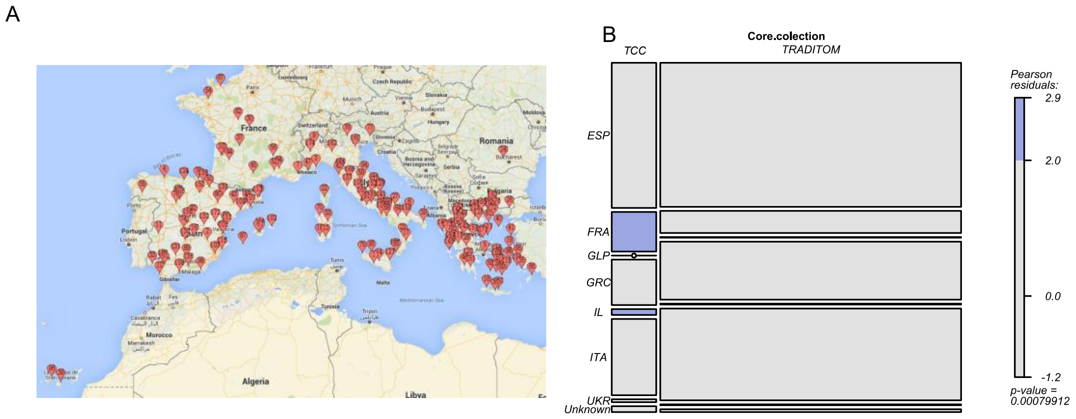

Supplementary Figure S2. Original collection sites of the European traditional European tomato accessions. (A) Data represent 1472 accessions with passport data in the original collection aggregated on to the level of province. (B) Mosaic plot showing the distribution of accessions from different countries in the TCC and in the TRADITOM collection. Enrichment was evaluated by departure of residuals from the expected value. Residuals with |dij|> 4 have an approximate P-value < 0.001 and |dij|> 2 have an approximate P-value < 0.05. The Chi-square (χ^2) independence test results for distribution comparison is shown.

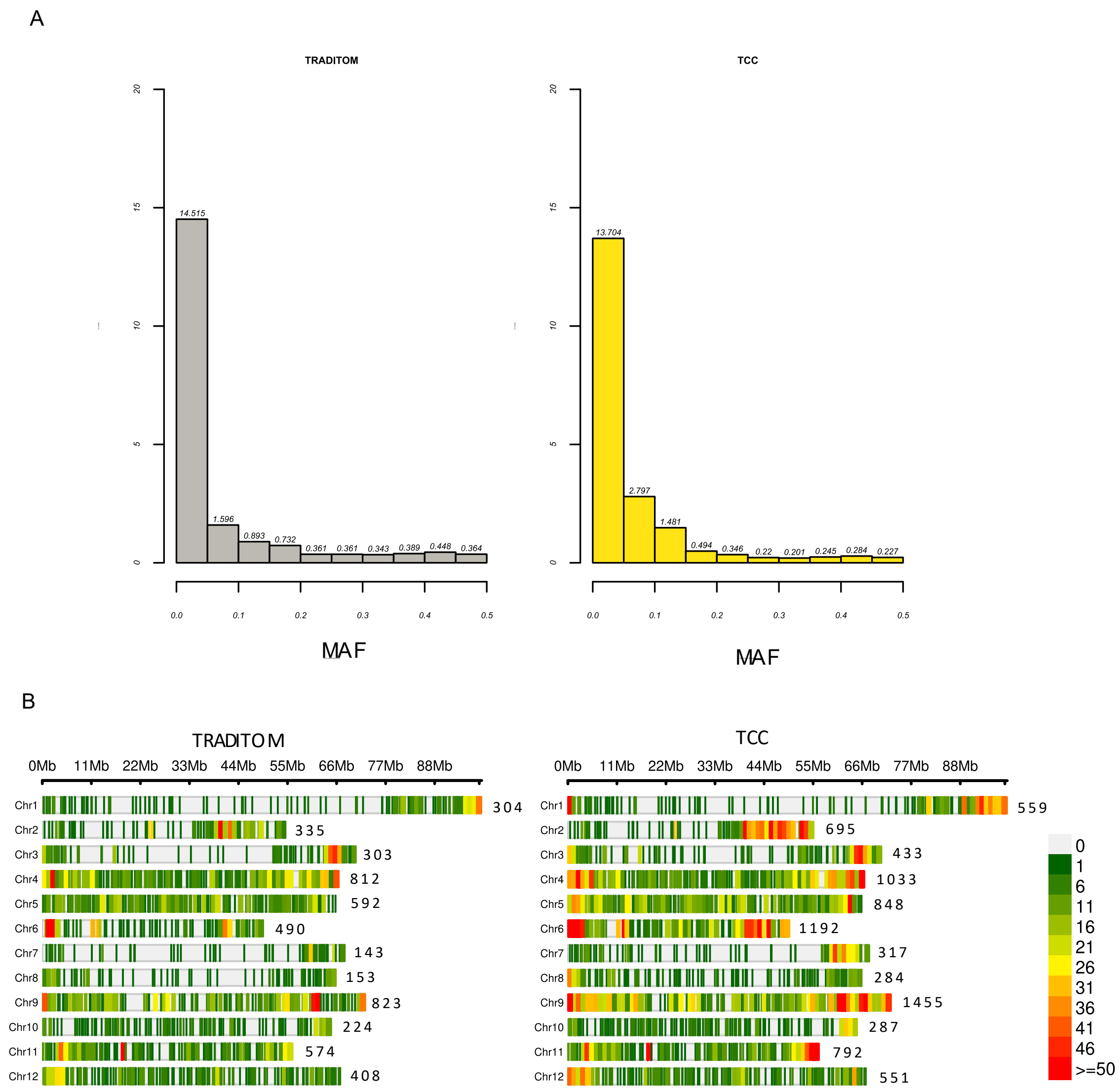

Supplementary Figure S3. SNP distribution in TRADITOM and in the TCC collections. (A) Histogram of the SNP with Minor allele frequency (MAF) ≥0.01 in TRADITOM collection (left panel) and in the TCC collection (right panel). (B) SNP density plot chromosome wise representing number of SNPs with MAF > 0.01 within 1 Mb window size along TRAIDTOM collection (A) and TCC collection (B). The horizontal axis shows the chromosome length (Mb); the different color depicts SNP density; the number of SNPs per chromosome is indicated at the right side o each chromosome. Chromosome chr00, representing unplaced sequences is not plotted

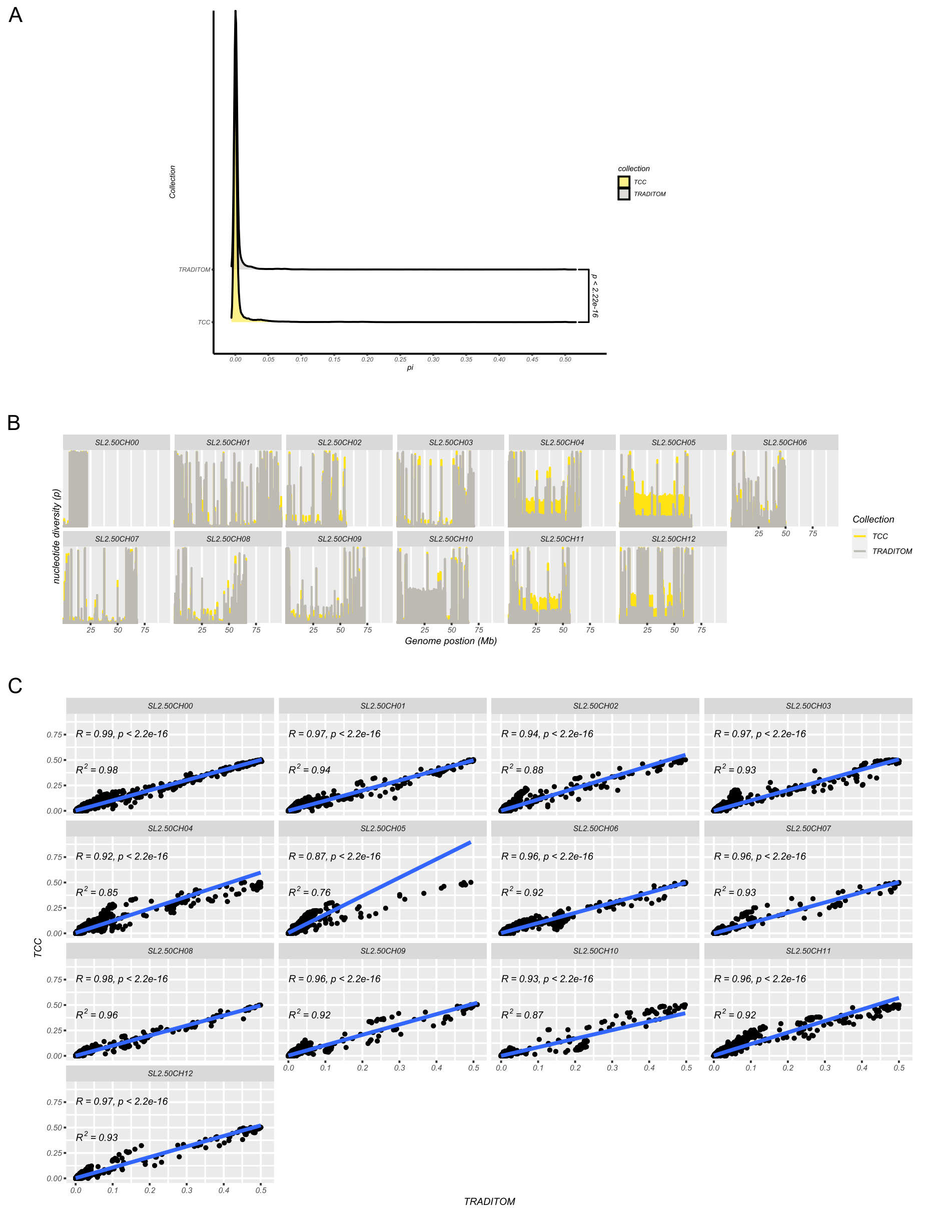

Supplementary Figure S4. Comparison of nucleotide diversity (π) between TRADITOM and the TCC collections. (A) Nucleotide diversity (π) distribution of the 110909 SNPs in the TRAIDTOM and the TCC. The distributions are presented as densities. The p-value of the ANOVA test comparing distributions is shown. (B) Nucleotide diversity (π) plot chromosome along TRAIDTOM collection and TCC collection (C). Correlation between the nucleotide diversity per chromosoe in TRADITOM and the TCC collections The horizontal axis shows TRADITOM π values and the vertical axis, the π of the TCC. Squared correlation (R^2) and p-value of correlation is shown for each chromosome.

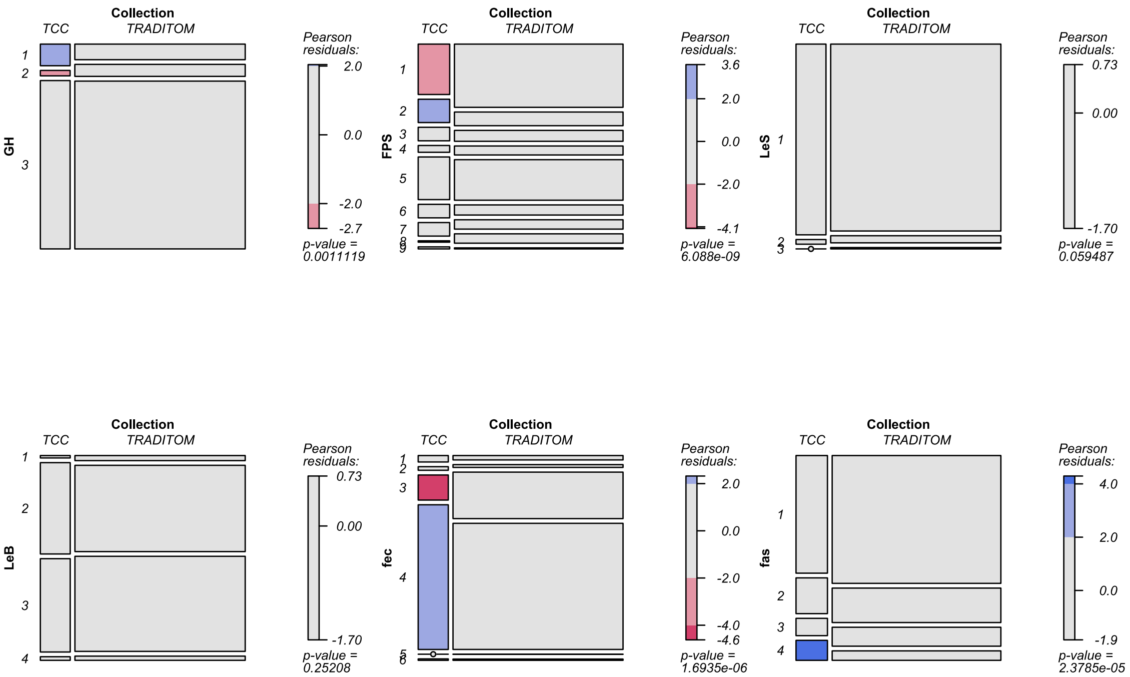

Supplementary Figure S5. Comparison of quantitative trait frequency distribution in the TCC and the TRADITOM collections. Mosaic plots showing the frequencies and enrichment of trait categories in six selected quantitative traits. Growth habit (GH), Fruit predominant shape (FPS), Leaf Shape (LeS), Leaf Border (LeB), fruit external colour (FEC) and fruit fasciation (FAS). Enrichment was evaluated by departure of residuals from the expected value. Residuals with |dij|> 4 have an approximate P-value < 0.001 and |dij|> 2 have an approximate P-value < 0.05. The Chi-square (χ^2) independence test results between TRADITOM and TCC are shown. The score of each trait is: GH 1: determinate, 2: indeterminate, 3:determinate; FPS 1: flat, 2: rectangular, 3: ellipsoid, 4: obovoid, 5: round, 6: oxheart, 7: long, 8: heart, 9: Bell pepper; LeS 1: regular leaf, 2: potato leaf, 3: double feathered; LeB 1: entire, 2: undulate, 3: serrated, 4: strong serrated; FEC 1: yellow, 2: orange, 3: pink, 4: red, 5: purple, 6: brown, 7: green; FAS 1: not present, 2: low, 3: intermediate, 4: severe

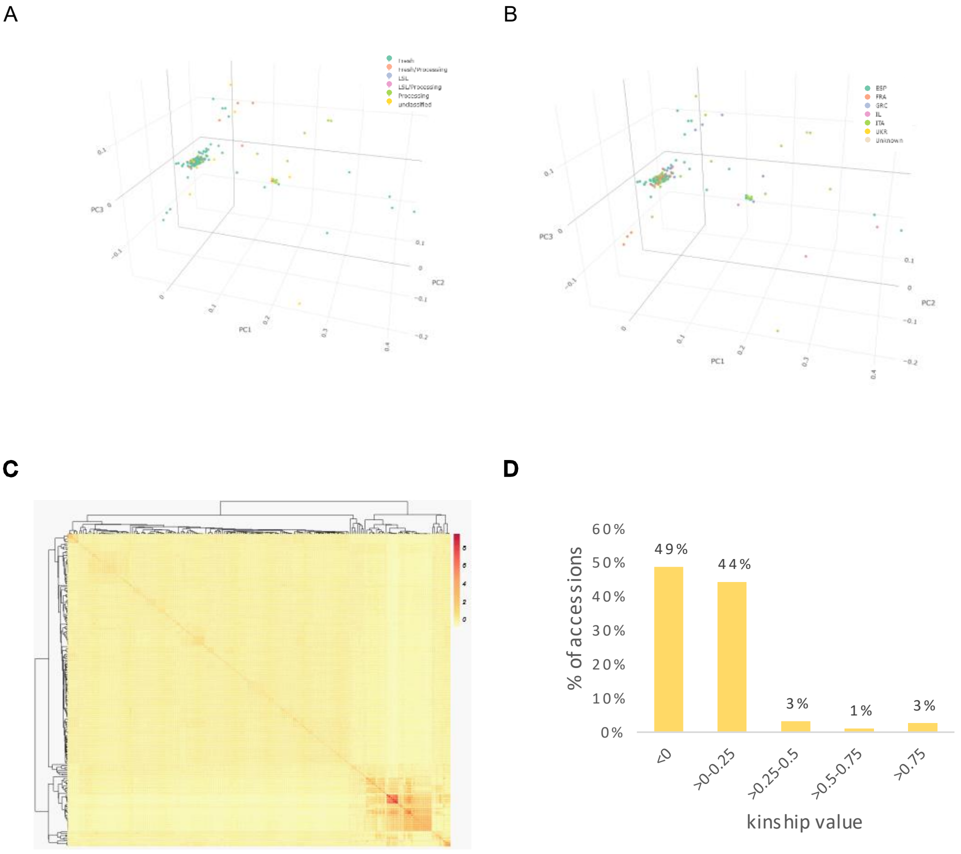

Supplementary Figure S6. Population structure and kinship. MDS and kinship are derived from 225 accessions from the TCC collection using SNPs with MAF>0.02. Kinship Analysis of the. The population structure based on the end use (A) and the country of origin. (C) Kinship matrix showing the relatedness of the tomato accessions in the TCC. The bar colour depicts the kinship value. (D) Histogram showing the distribution of the kinship value in the TCC.

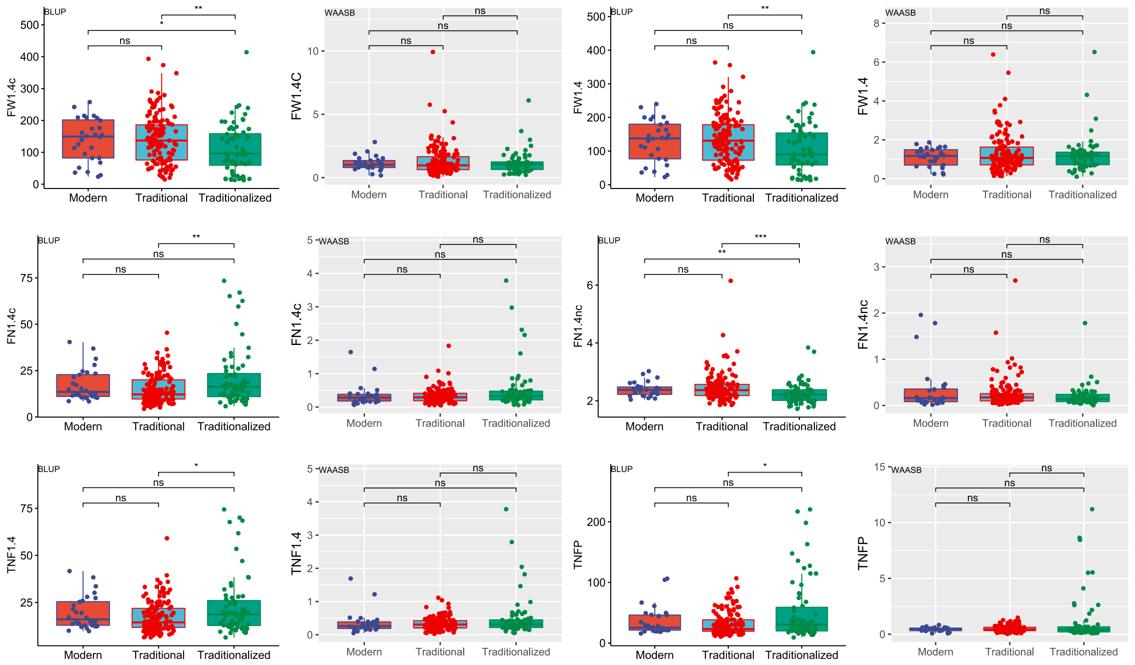

Supplementary Figure S7. Boxplot showing the differences in the genotypic mean and the stability of traits related to fruit weight and fruit number among the three tomato groups. Dots represent the genotypic BLUP or WAASB index for each accession. The line within the box indicates the median. Box edges indicate the 25-75th percentile for each trait. Error bars indicate the 5th and 95th percentiles. Comparisons between different tomato groups with asterisk *, **, ***, **** are statistically different at probability values of $p \leq 0.05$, ≤ 0.01, ≤ 0.001 and ≤ 0.0001, respectively.

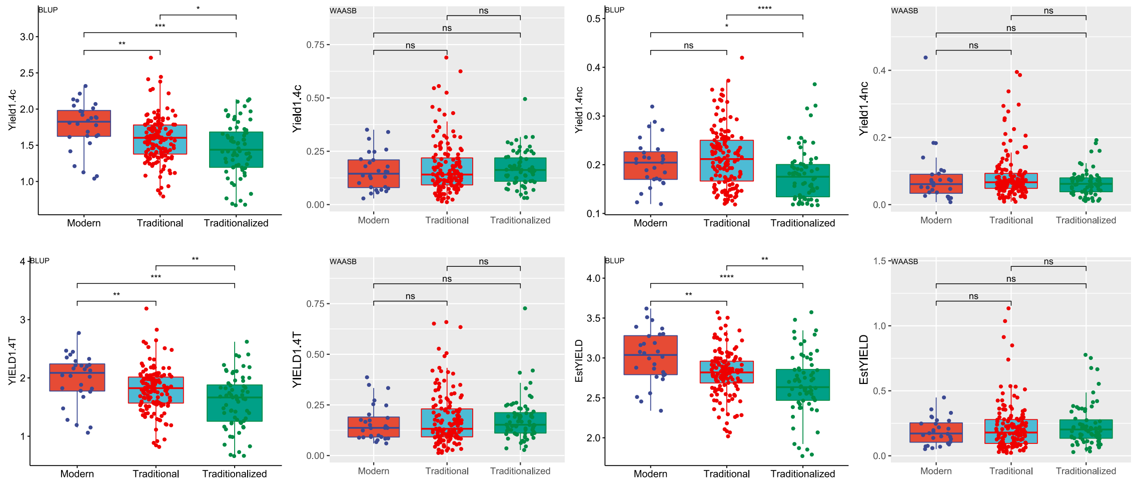

Supplementary Figure 8. Boxplot showing the differences in the genotypic mean and the stability of traits related to fruit yield among the three tomato groups. Dots represent the genotypic BLUP or WAASB index for each accession. The line within the box indicates the median. Box edges indicate the 25-75th percentile for each trait. Error bars indicate the 5th and 95th percentiles. Comparisons between different tomato groups with asterisk *, **, ***, **** are statistically different at probability values of $p \leq 0.05$, ≤ 0.01, ≤ 0.001 and ≤ 0.0001, respectively.

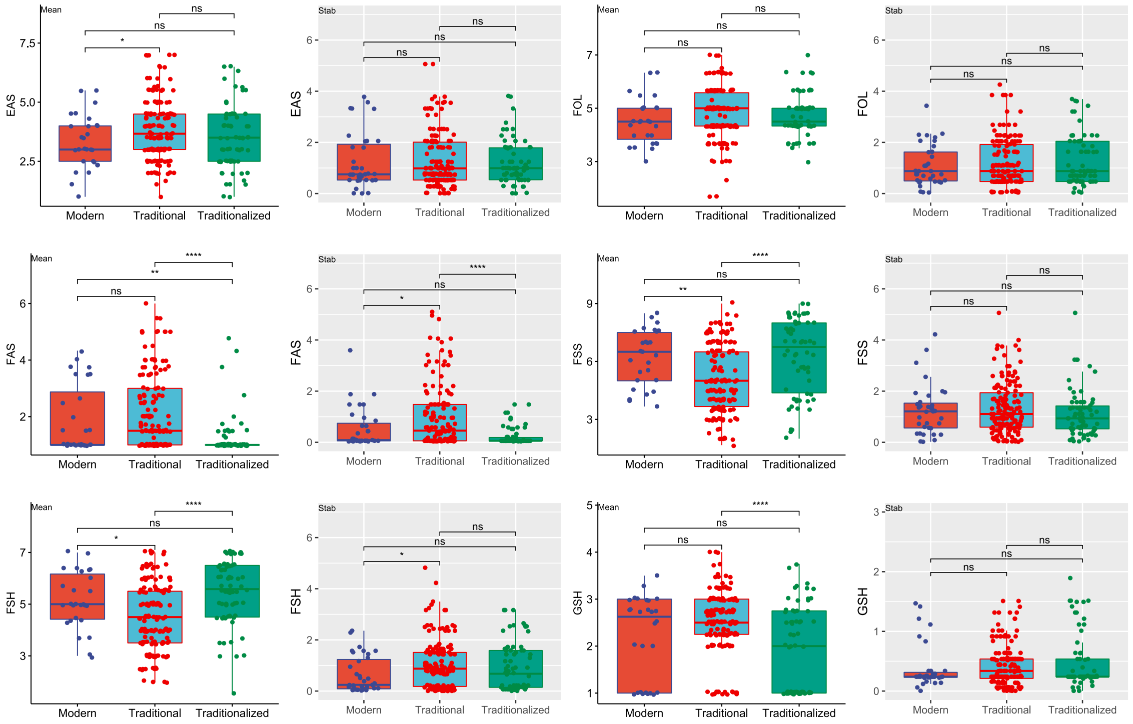

Supplementary Figure S9 Boxplot showing the differences among the three tomato groups in the genotypic mean and the stability of easiness to detach from the pedicel, foliage density, fasciation, fruit set sequence, fruit size homogeneity and green shoulder. Dots represent the genotypic BLUP or WAASB index for each accession. The line within the box indicates the median. Box edges indicate the 25-75th percentile for each trait. Error bars indicate the 5th and 95th percentiles. Comparisons between different tomato groups with asterisk *, **, ***, **** are statistically different at probability values of $p \leq 0.05$, ≤ 0.01, ≤ 0.001 and ≤ 0.0001, respectively.

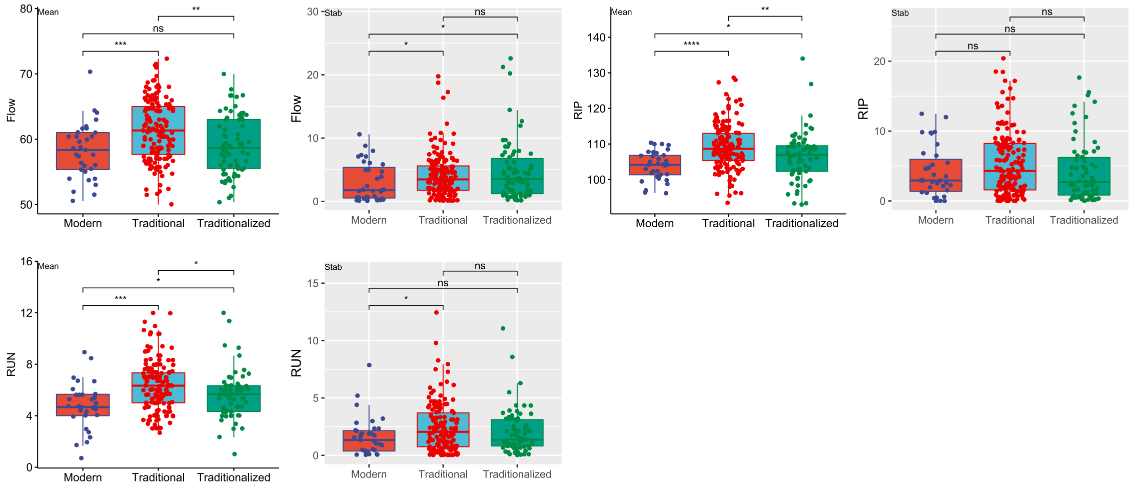

Supplementary Figure 10. Boxplot showing the differences in the genotypic mean and the stability of traits related to flowering and ripening precocity. Dots represent the genotypic BLUP or WAASB index for each accession. The line within the box indicates the median. Box edges indicate the 25-75th percentile for each trait. Error bars indicate the 5th and 95th percentiles. Comparisons between different tomato types with asterisk *, **, ***, **** are statistically different at probability values of $p \leq 0.05$, ≤ 0.01, ≤ 0.001 and ≤ 0.0001, respectively.

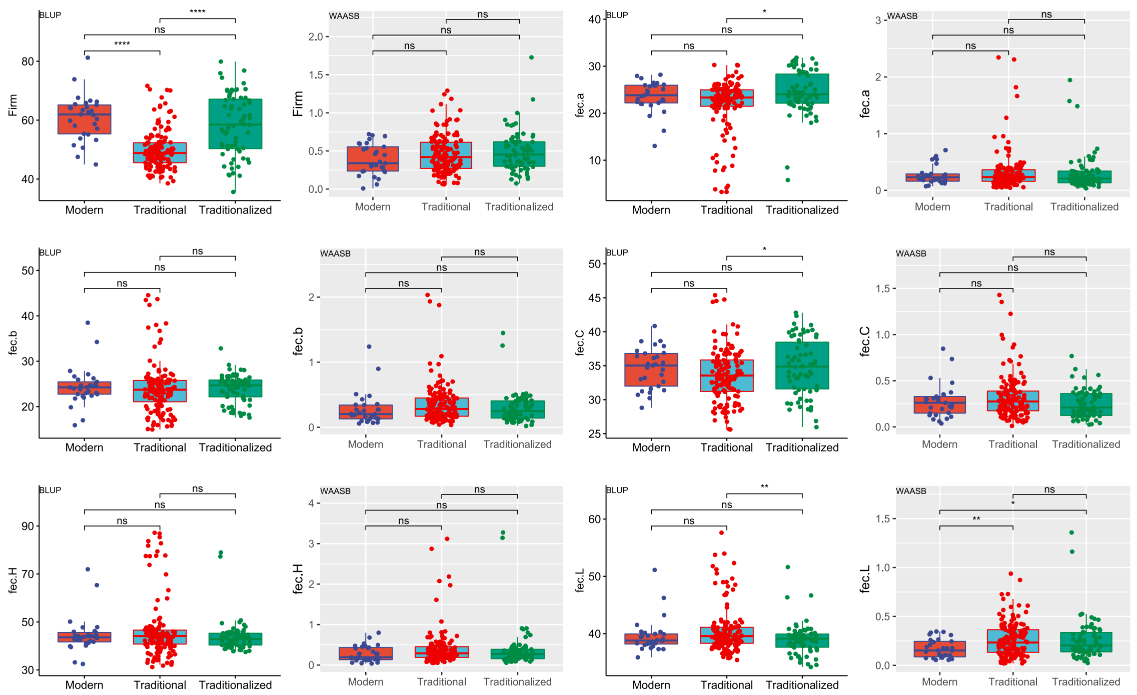

Supplementary Figure S11. Boxplot showing the differences in the genotypic mean and the stability of traits related to fruit quality among the three tomato groups. Dots represent the genotypic BLUP or WAASB index for each accession. The line within the box indicates the median. Box edges indicate the 25-75th percentile for each trait. Error bars indicate the 5th and 95th percentiles. Comparisons between different tomato groups with asterisk *, **, ***, **** are statistically different at probability values of p ≤ 0.05, ≤ 0.01, ≤ 0.001 and ≤ 0.0001, respectively.

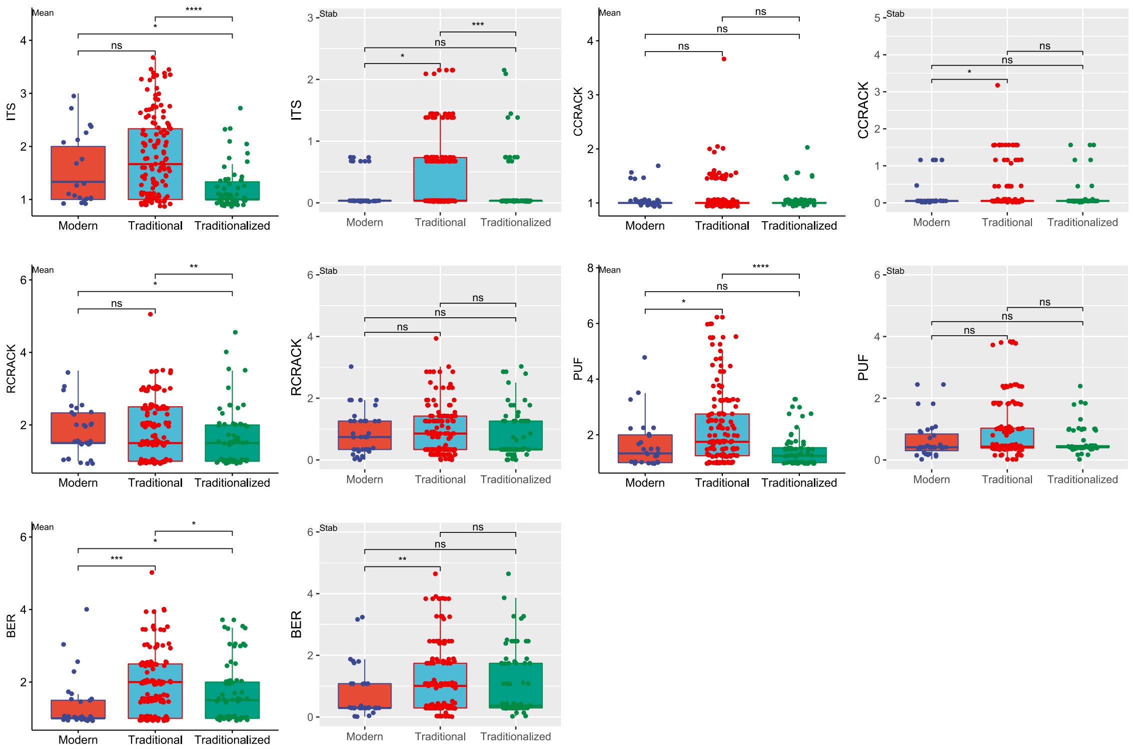

Supplementary Figure S12. Boxplot showing the differences among the three tomato groups in the genotypic mean and the stability of irregular transversal section, foliage density and physiological disorders. Dots represent the genotypic BLUP or WAASB index for each accession. The line within the box indicates the median. Box edges indicate the 25-75th percentile for each trait. Error bars indicate the 5th and 95th percentiles. Comparisons between different tomato groups with asterisk *, **, ***, **** are statistically different at probability values of $p \leq 0.05$, ≤ 0.01, ≤ 0.001 and ≤ 0.0001, respectively.

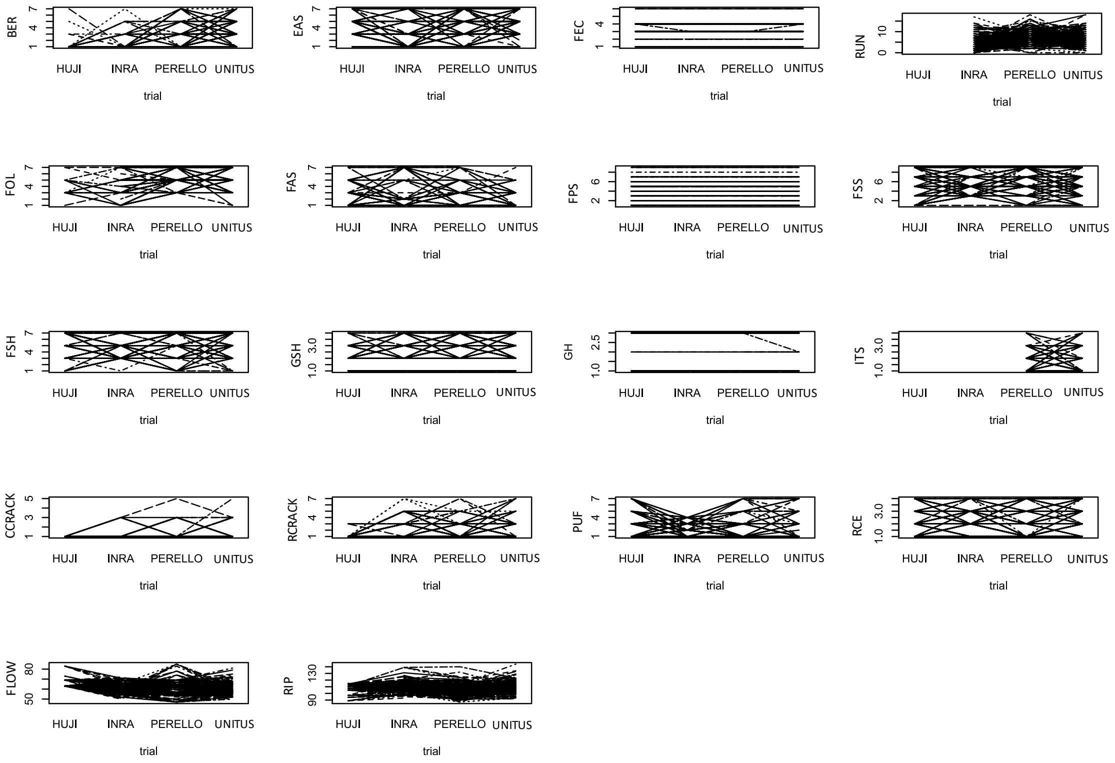

Supplementary Figure S13. Interaction plots of trait means for each accession across locations. X-axis represents the location, and the y- axis the mean value of the trait at each location.

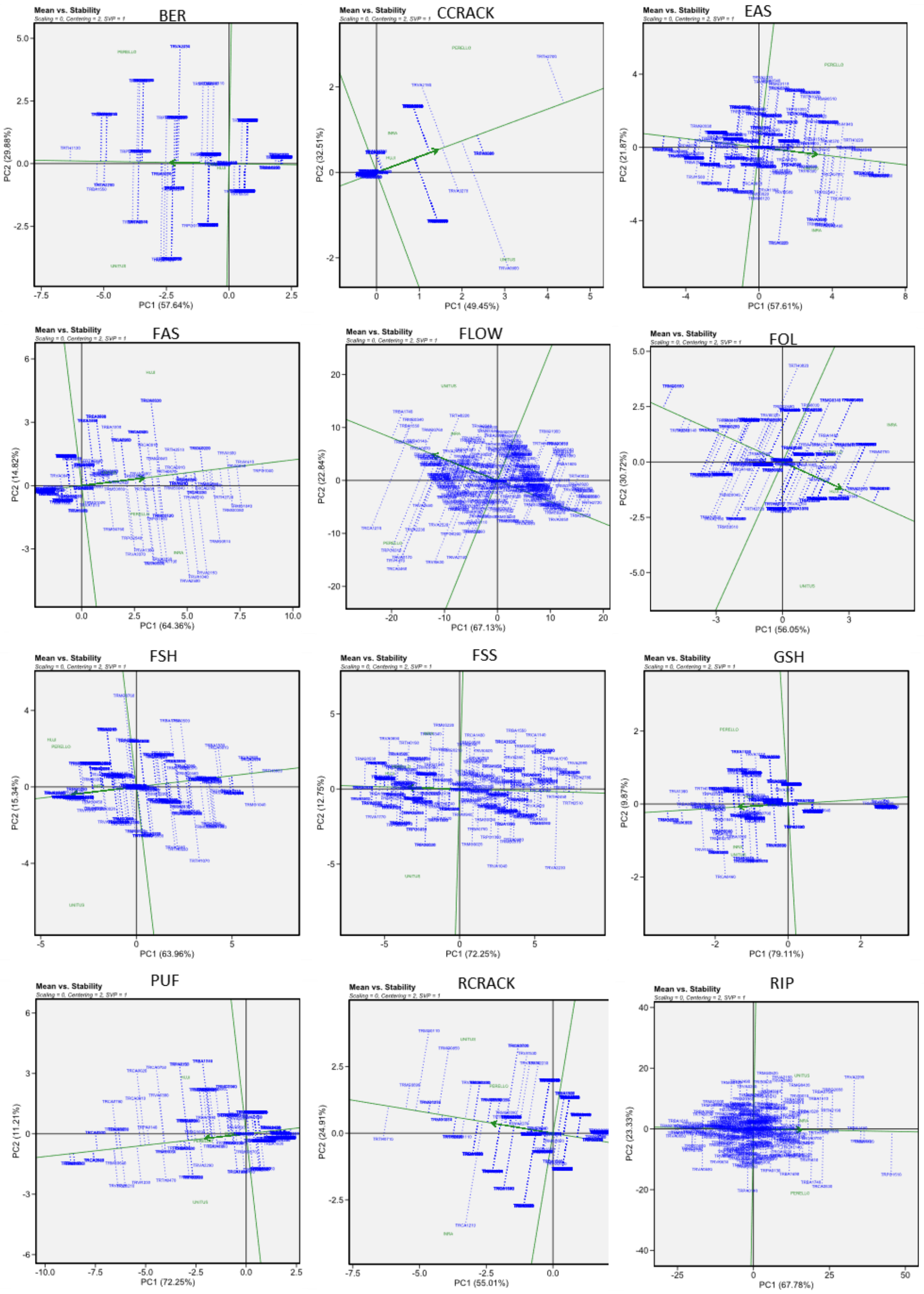

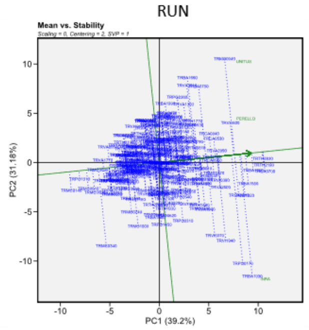

Supplementary Figure S14. Mean performance vs. stability GGE genotype-focused biplots for accession and location. GGE biplots constructed using the first two symmetrically scaled principal components (PC1 and PC2) derived from singular value decomposition (SVD) of environment- centred data. The position of each location in the PCA derived from SVD is coloured in green and the accessions in blue. Green lines represent the average environment coordinate (AEC) on the abscissa. The AEC abscissa represents the genotype effect, the AEC ordinate represents the GEI effect associated to each accession. The projection of each accession onto the AEC ordinate (dotted blue lines) represents the stability. A greater projection onto the AEC ordinate, lower stability. The name of the traits is indicated above the biplot.

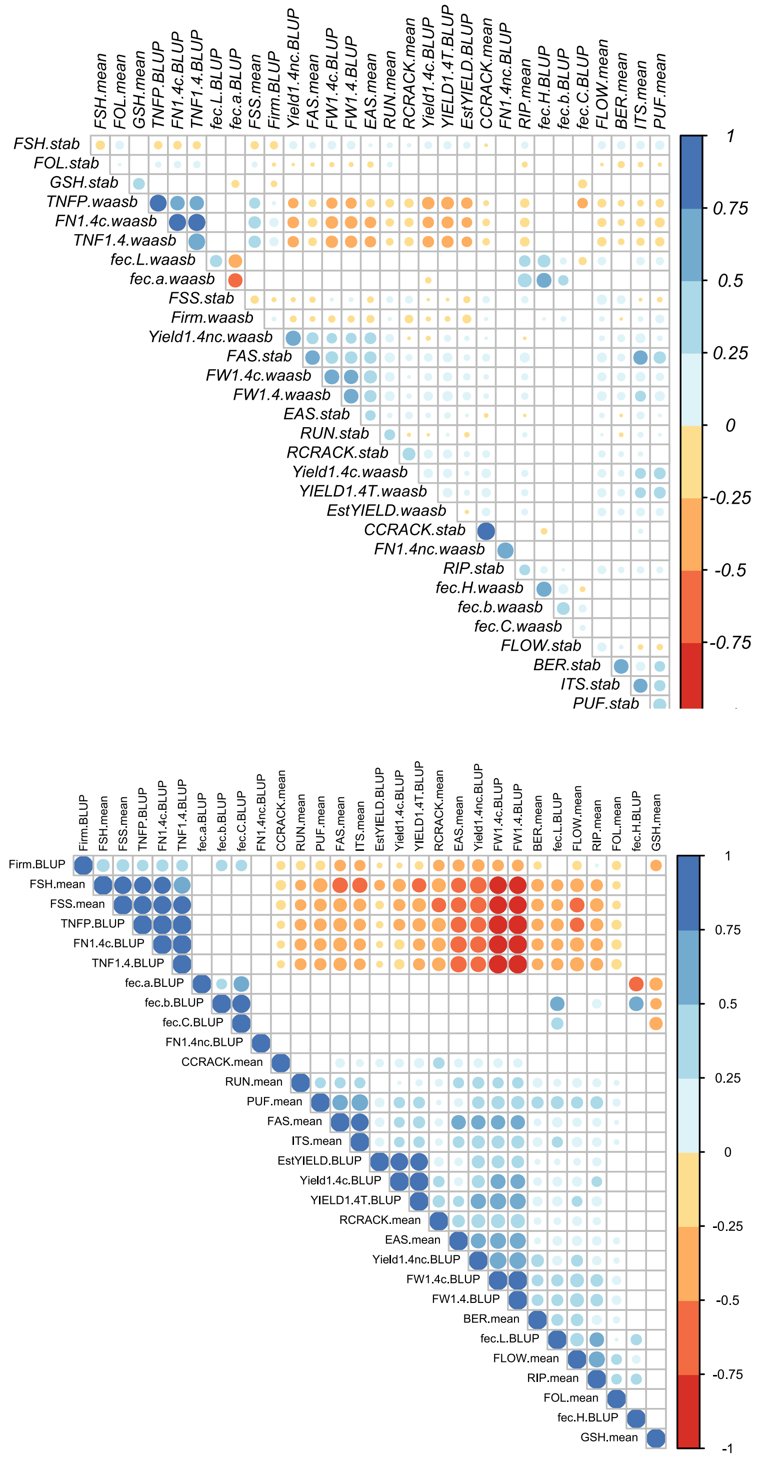

Supplementary Figure S15. Pearson's correlations across the different locations. A) Between the phenotypic means and stability index. **B)** Between the phenotypic means of 30 traits across the different locations Traits not showing significant GXE and E effect are omitted. Only significant correlations at p-value <0.01 are shown. Blue denotes positive correlations, and red negative correlations, BLUP, Best linear unbiased genotypic predictors; WASSB, Weighted Average of Absolute Scores; Stab, genotype projection onto de AEC vertical axis.

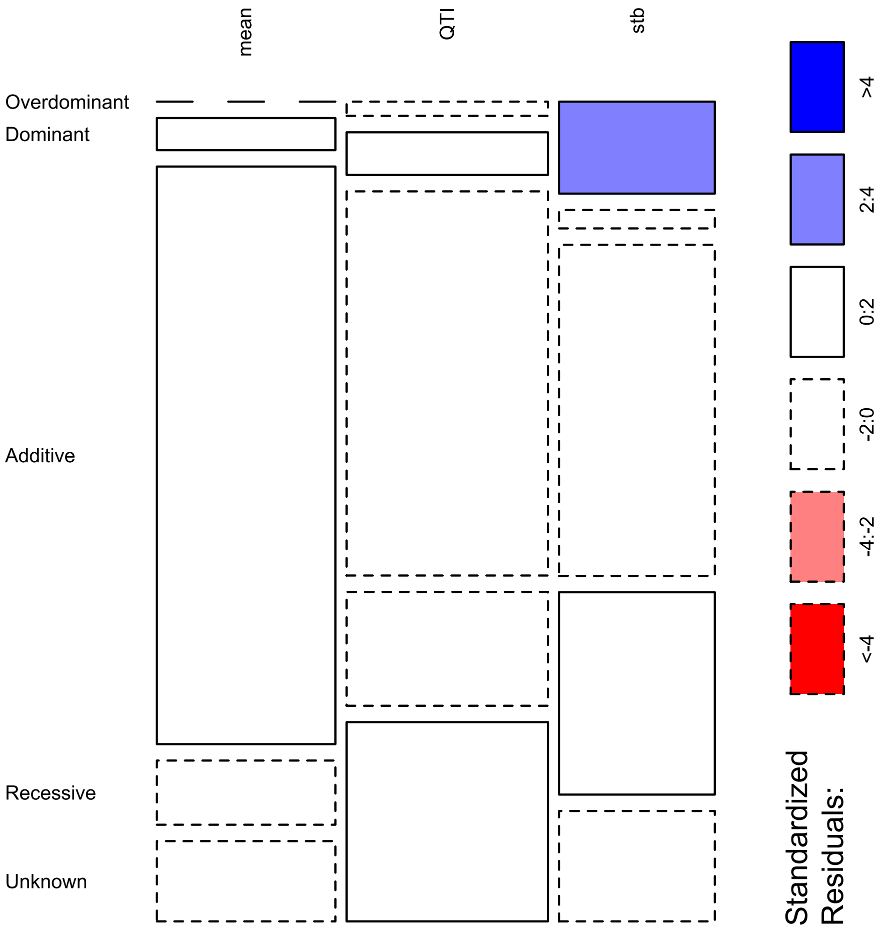

Supplementary Figure S16. Mosaic plots showing the distribution of each mode of inheritance among the three QTL classes (meanQTL, stbQTL, QTI). Enrichment of an inheritance mode within a QTL class was evaluated by departure of residuals from the expected value. Colour indicates the enrichment significance. Residuals with |dij|> 4 have an approximate P-value < 0.001 and |dij|> 2 have an approximate P-value < 0.05.

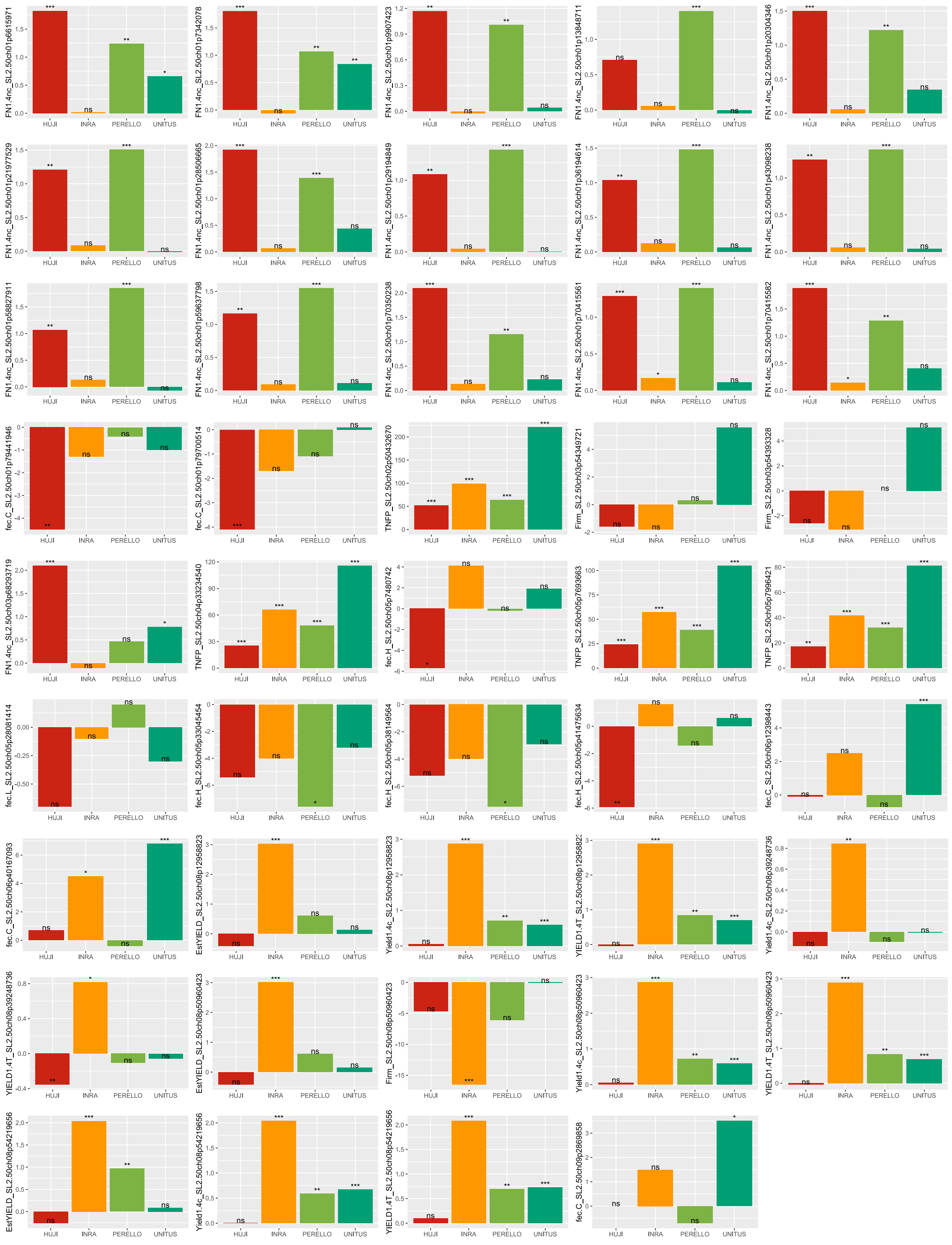
FN1.4nc_SL2.50ch01p6615971
FN1.4nc_SL2.50ch01p7342078
FN1.4nc_SL2.50ch01p9907423
FN1.4nc_SL2.50ch01p13848711
FN1.4nc_SL2.50ch01p20304346
FN1.4nc_SL2.50ch01p21977529
FN1.4nc_SL2.50ch01p28506665
FN1.4nc_SL2.50ch01p29194849
FN1.4nc_SL2.50ch01p36194614
FN1.4nc_SL2.50ch01p43098238
FN1.4nc_SL2.50ch01p58827911
FN1.4nc_SL2.50ch01p59637798
FN1.4nc_SL2.50ch01p70350238
FN1.4nc_SL2.50ch01p70415561
FN1.4nc_SL2.50ch01p70415582
fec.C_SL2.50ch01p79441946
fec.C_SL2.50ch01p79700514
TNFP_SL2.50ch02p50432670
Firm_SL2.50ch03p54349721
Firm_SL2.50ch03p54393328
FN1.4nc_SL2.50ch03p68293719
TNFP_SL2.50ch04p33234540
fec.H_SL2.50ch05p7480742
TNFP_SL2.50ch05p7693663
TNFP_SL2.50ch05p7996421
fec.L_SL2.50ch05p28081414
fec.H_SL2.50ch05p33045454
fec.H_SL2.50ch05p38149564
fec.H_SL2.50ch05p41475634
fec.C_SL2.50ch06p12398443
fec.C_SL2.50ch06p40167093
EstYIELD_SL2.50ch08p12958823
Yield1.4c_SL2.50ch08p12958823
YIELD1.4T_SL2.50ch08p1295882
Yield1.4c_SL2.50ch08p39248736
YIELD1.4T_SL2.50ch08p39248736
EstYIELD_SL2.50ch08p50960423
Firm_SL2.50ch08p50960423
Yield1.4c_SL2.50ch08p50960423
YIELD1.4T_SL2.50ch08p50960423
EstYIELD_SL2.50ch08p54219656
Yield1.4c_SL2.50ch08p54219656
YIELD1.4T_SL2.50ch08p54219656
fec.C_SL2.50ch09p2869858
HUJI
INRA
PERELLO
UNITUS

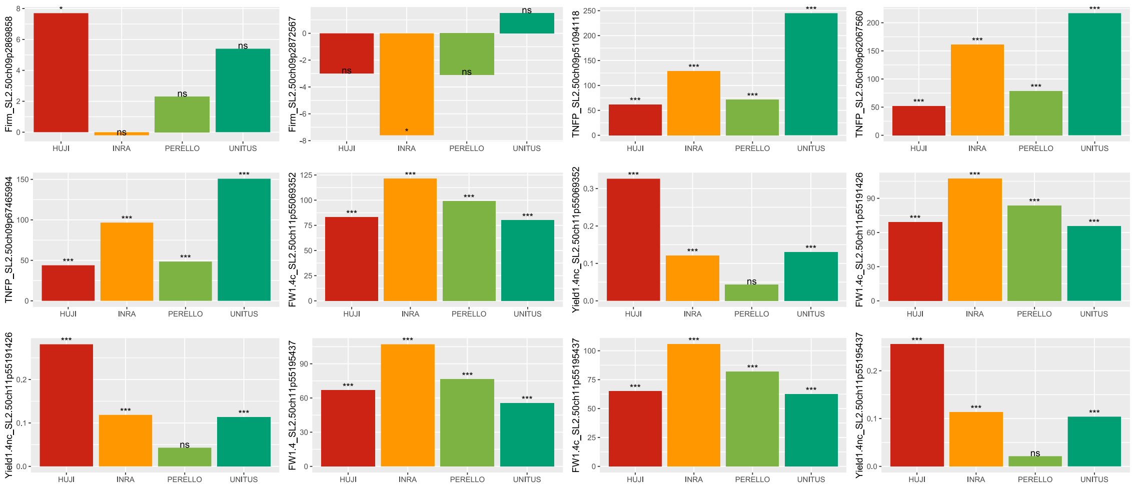

Supplementary Figures S17. Location-specific allelic effects of identified of MTAs in identified QTIs. Each graphic represents the difference of the marginal means of BLUPge for the two homozygous marker allelic states at each location and for each trait-marker association. Comparisons between different location-specific allelic effects labelled with asterisk *, **, ***, **** are statistically different at probability values of p ≤ 0.05, ≤ 0.01, ≤ 0.001 and ≤ 0.0001, respectively.

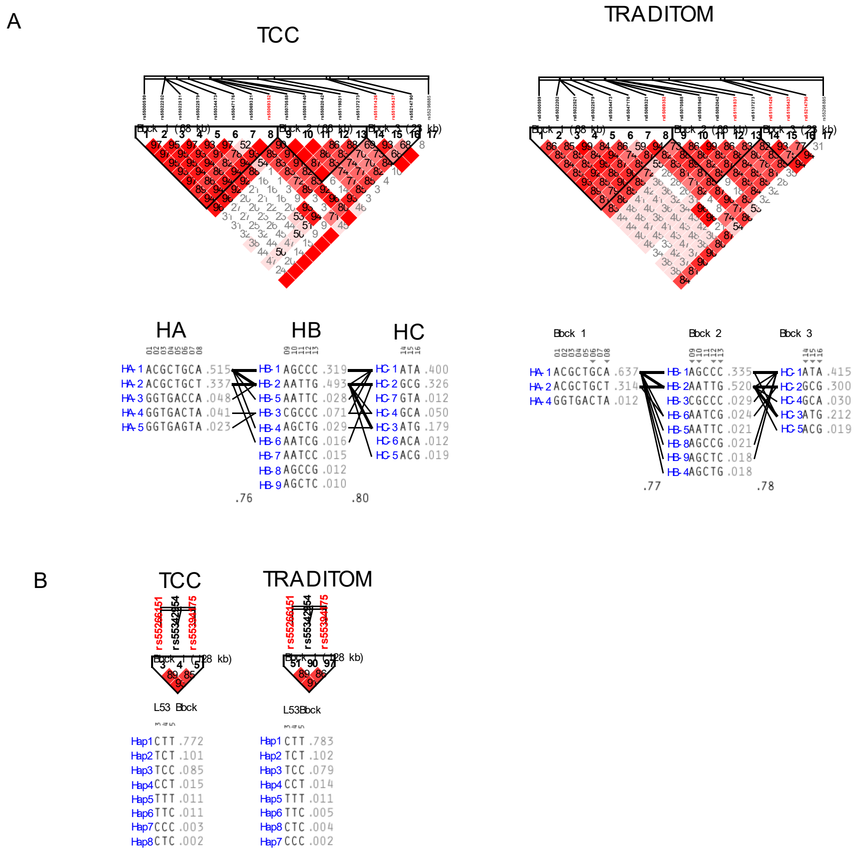

Supplementary Figure S18. Linkage Disequilibrium (LD) and Haplotype Analysis of loci TCC_L62 and TTC_L53 in the TCC and TRADITOM collection. (A) LD plots and haplotype block structure across the TCC_L62 locus containing the GWAS identified SNP for fruit weight in the TCC (left panel) and in the entire TRADITOM collection (right panel). (B) LD plots and haplotype block structure across the TCC_L53 locus containing the GWAS identified SNP for fruit weight in the TCC (left panel) and in the entire TRADITOM collection (right panel).

The upper diagram shows the relative physical position of each SNP. GWAS identified SNPs are highlighted in red. Blocks depict the estimated D' (Lewontin's normalized LD) between pair of markers. Red blocks, *D'*> 0.8 with LOD (log of the likelihood odds ratio) of 2, white blocks, *D'* < 1.0 with LOD < 2.0. D prime values of 1.0 are never shown. Haplotype blocks are delimited by black triangles within the heatmap. The lower diagram depicts common haplotypes (>1%) and estimated haplotype frequencies in each haplotype block. Haplotype frequency is shown to the right of each haplotype. Only haplotypes with frequencies higher than 1% are shown. In the crossing areas, a value of multiallelic D' (estimation of the level of recombination between two blocks) is shown. Connections from one block to the next are shown for haplotypes with a frequency >10% as thick lines and a frequency >1% as thin lines.

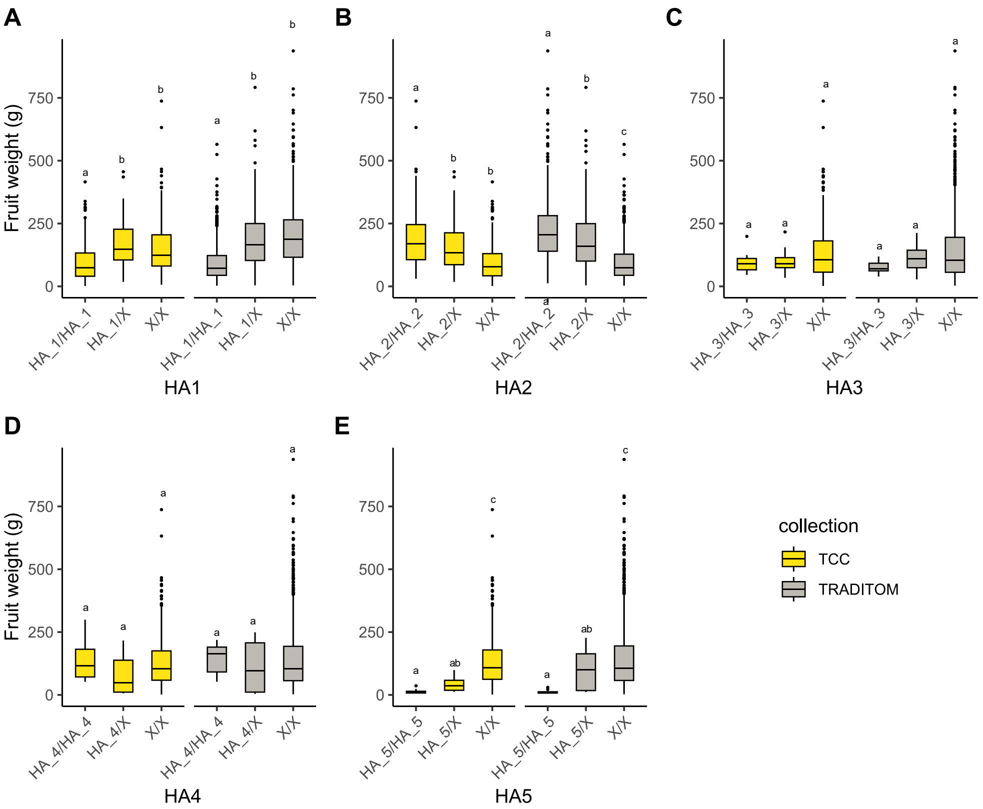

Figure S19. Association between absence (0 copies) and presence (1 or 2 copies) of block TCC_L62 HA haplotypes on *FW* in the TCC and TRADITOM collection. Absence of target haplotype is coded by "X" haplotype. Boxplots show the of different haplotypes HA block on *FW*. Each panel corresponded to one haplotype (A) HA1, (B) HA2, (C) HA3, (D) HA4 and(E) HA5. Different letters above box-plots indicate statistically significant differences ($p<0.05$). X axis depicts the haplotype and Y axis the *FW*. The horizontal bar inside the box-plot indicate the mean value. Bars in box-plot represent standard deviation, and dots, corresponded to outliers.

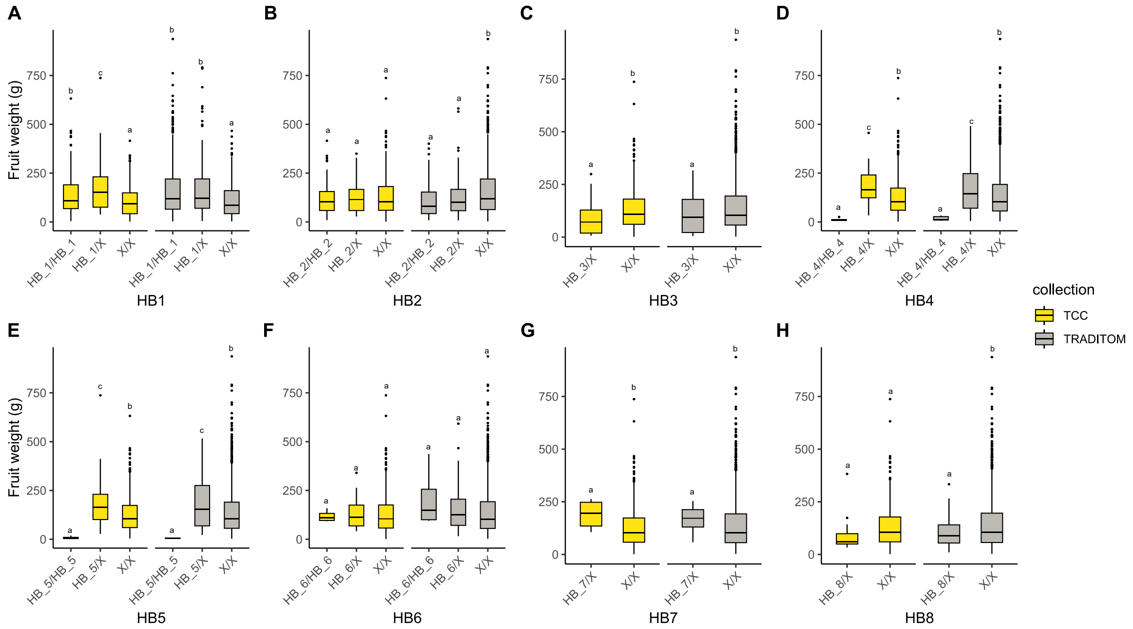

Figure S20. Association between absence (0 copies) and presence (1 or 2 copies) of block TCC_L62 HB haplotypes on *FW* in the TCC and TRADITOM collection. Absence of target haplotype is coded by “X” haplotype. Boxplots show the of different haplotypes HB block on *FW*. Each panel corresponded to one haplotype (A) HB1, (B) HB2, (C) HB3, (D) HB4, (E) HB5, (F) HB6, (G) HB7 and (H) HB5. Different letters above box-plots indicate statistically significant differences ($p<0.05$). X axis depicts the haplotype and Y axis the *FW*. The horizontal bar inside the box-plot indicate the mean value. Bars in box-plot represent standard deviation, and dots, corresponded to outliers.

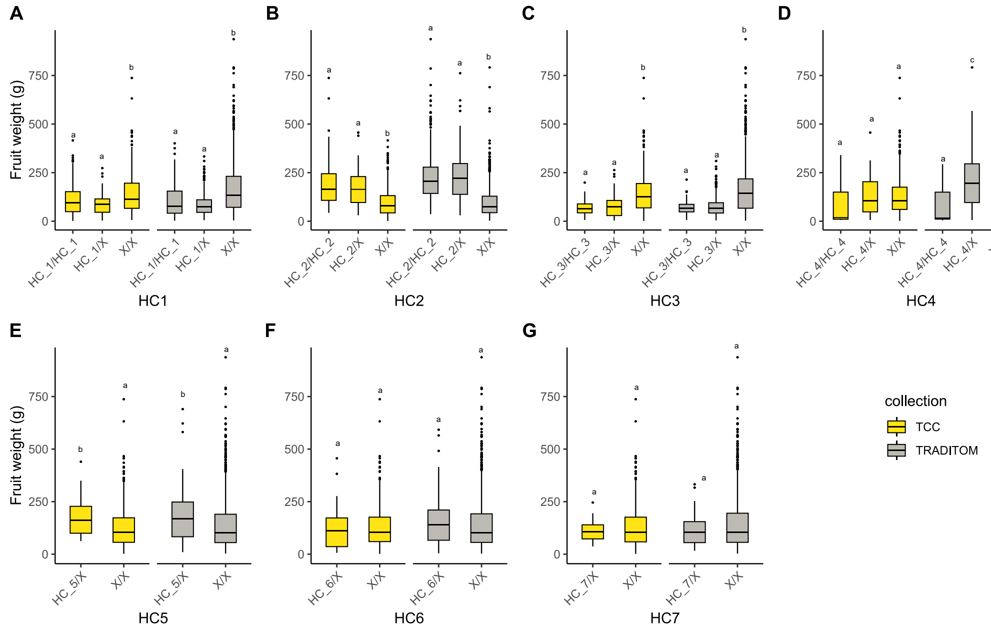

Figure S21. Association between absence (0 copies) and presence (1 or 2 copies) of block TCC_L62 HC haplotypes on *FW* in the TCC and TRADITOM collection. Absence of target haplotype is coded by "X" haplotype. Boxplots show the of different haplotypes HC block on *FW*. Each panel corresponded to one haplotype (A) HC1, (B) HC2, (C) HC3, (D) HC4, (E) HC5, (F) HC6 and (G) HC7. Different letters above box-plots indicate statistically significant differences ($p<0.05$). X axis depicts the haplotype and Y axis the *FW*. The horizontal bar inside the box-plot indicate the mean value. Bars in box-plot represent standard deviation, and dots, corresponded to outliers.

.

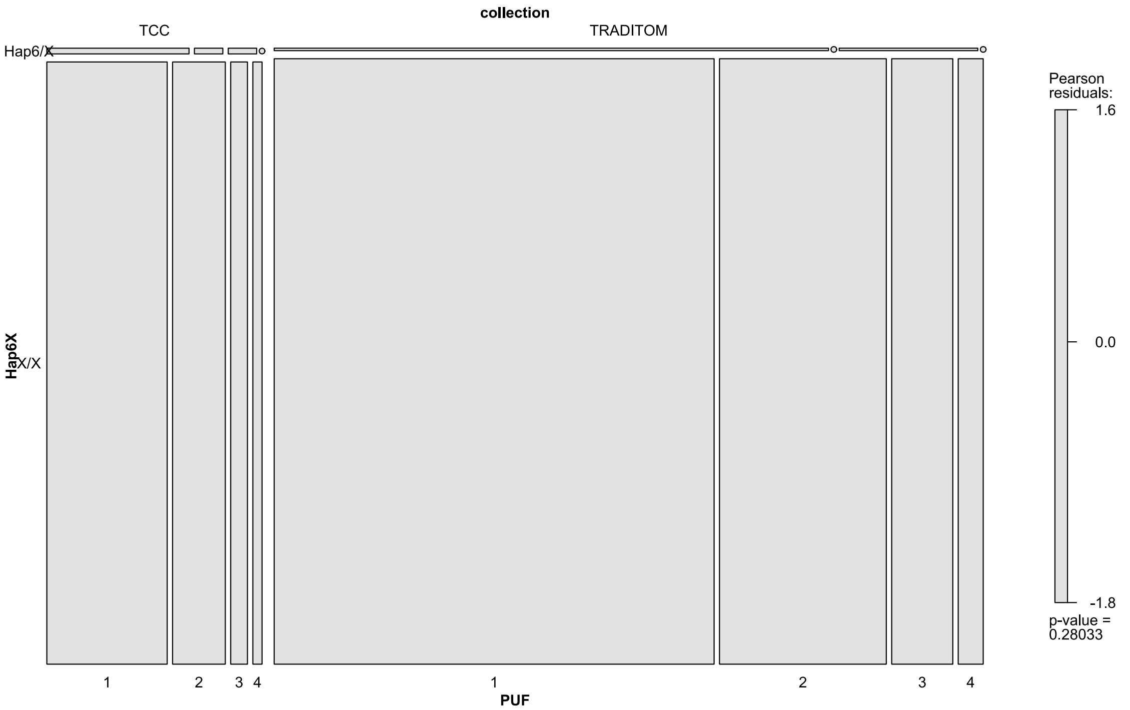

Figure S22: Association between absence (0 copies) and presence (1 or 2 copies) of TCC_L53 locus hap6 haplotype on PUF in the TCC and TRADITOM collection Mosaic plots show the effect of the presence/absence for **TCC_L53 locus hap6 haplotype on** PUF degree. The colour of the mosaic depicts the enrichment, evaluated by departure of Pearson residuals (dij) from the expected value. Residuals with |dij|> 4 have an approximate P-value < 0.001 and |dij|> 2 have an approximate P-value < 0.05. Yellow colour indicates over-representation and red under-representation.
